# Rhythmic auditory cueing in atypical parkinsonism: A pilot study

**DOI:** 10.3389/fneur.2022.1018206

**Published:** 2022-10-28

**Authors:** Alexander Pantelyat, Gabriel Dayanim, Kyurim Kang, Bela Turk, Ruben Pagkatipunan, Sera-Kim Huenergard, Albert Mears, Jee Bang

**Affiliations:** ^1^Department of Neurology, Johns Hopkins University School of Medicine, Baltimore, MD, United States; ^2^Center for Music and Medicine, Johns Hopkins University School of Medicine, Baltimore, MD, United States; ^3^College of Computer, Mathematical, and Natural Sciences, University of Maryland, College Park, MD, United States; ^4^Departments of Neurology and Pediatrics, Moser Center for Leukodystrophies, Kennedy Krieger Institute, Johns Hopkins Medical Institutions, Baltimore, MD, United States; ^5^Department of Rehabilitation, Johns Hopkins Bayview Medical Center, Baltimore, MD, United States

**Keywords:** atypical parkinsonian disorders, progressive supranuclear palsy (PSP), corticobasal syndrome (CBS), multiple system atrophy (MSA), rhythmic auditory stimulation (RAS), Rhythmic auditory cueing

## Abstract

Rhythmic auditory cueing (RAC) can improve gait parameters in neurological disorders such as Parkinson's disease and stroke. However, there is a lack of research on the effects of RAC in patients with atypical parkinsonian disorders (APD). Using a smartphone metronome application, we aimed to investigate the immediate effects of RAC in patients with clinically diagnosed APD, namely Progressive Supranuclear Palsy (PSP-Richardson Syndrome and other variants, PSP-nonRS), Corticobasal Syndrome (CBS), Multiple System Atrophy (MSA), and Dementia with Lewy Bodies (DLB). A total of 46 APD participants (25 PSP, 9 CBS, 8 MSA and 4 DLB; age: mean = 70.17, standard deviation = 7.15) walked at their preferred pace for 2 min without any rhythmic auditory cueing (RAC). Participants then walked the same path for another 2 min with RAC set at a tempo 10% faster than the baseline cadence of each participant. After a 10–15-min break, participants walked the same path for another 2 min without RAC to observe for carryover effects. Gait parameters [cadence (steps/minute), gait velocity (meters/minute), and stride length (centimeters)] were collected at baseline, during RAC, and post-RAC. There was a significant improvement in cadence in all participants from baseline to during RAC and post-RAC (corrected *p*-values = 0.009 for both). Gait velocity also improved from baseline to during RAC and post-RAC in all participants, although this improvement was not significant after correcting for multiple comparisons. The changes in cadence and gait velocity were most pronounced in PSP. In addition, our exploratory analysis showed that the cadence in the suspected TAU group (PSP+CBS) showed a significant improvement from baseline to during RAC and post-RAC (corr. *p*-value = 0.004 for both). This pilot study using short-term RAC in APD patients demonstrated improvements in cadence and velocity. There is an urgent need for effective gait rehabilitation modalities for patients with APD, and rhythmic cueing can be a practical and useful intervention to improve their gait pattern.

## Introduction

A large body of literature supports the use of music and rhythm-based interventions to improve gait and aspects of quality of life in patients with neurological disorders [see reviews: (1, 2)]. Gait parameters in particular, have been shown to improve in patients with Parkinson's disease (PD), following these interventions.

Rhythmic auditory stimulus (RAS) is a Neurologic Music Therapy (NMT) technique that utilizes an auditory rhythmic cue to entrain gait to a specific rhythm (3). RAS, as an anticipatory time cue, can be used as both an immediate entrainment stimulus, providing rhythmic cues during movement, and as a facilitating stimulus for planning and executing a movement to achieve more functional gait patterns (3). There is evidence that different auditory stimuli may have different therapeutic effects, but an isochronous rhythmic stimulus structure (i.e., equal intervals between purses or beats) has been suggested as the best therapeutic intervention (4). Brain imaging studies have shown that auditory cues activate neural networks in multiple parts of the brain, including cortical, subcortical, brain stem, and cerebellum (5). Researchers have also found that auditory priming affects neural motor synchrony, mainly in beta oscillations in the supplementary motor area and the cerebellum (6, 7).

RAS is traditionally implemented by identifying the patient's baseline cadence first and then providing rhythmic cues using a metronome and/or live music at a faster or slower frequency (typically 5–10% increments) in order to improve the patient's gait. Cadence, gait velocity, and stride length are the commonly used parameters to monitor changes in a patient's gait (3, 8). All of these gait parameters have been successfully increased in PD patients by implementing RAS at 5–10% above baseline cadence (9–13). While music, which integrates both melody and rhythm, may be used, a simple monotone metronome beat has been effectively used as a rhythmic cue in patients with PD (14). In idiopathic PD, RAS has been shown to effectively improve gait velocity, cadence, stride length, and potentially decrease falls (9, 10, 15–18). These effects have been observed during RAS and documented gait improvements have lasted between 15 minutes and 6 months (11, 19, 20).

Since each patient has a unique preferred baseline cadence, RAS is implemented in an individualized manner for each patient instead of using a fixed metronome speed for all patients (17, 21). Responses to RAS may also be impacted by the severity of the patient's gait impairment and their baseline rhythmic abilities (22–24). For example, patients with particularly slow baseline cadences and prior musical experience have been shown to be more likely to benefit from RAS (22).

The effects of RAS on patients with atypical parkinsonian disorders (APD) remain largely unexplored. This study aimed to test the effects of rhythmic auditory cueing (RAC) using a metronome on patients with APD. We use the term RAC rather than RAS in this paper because the intervention was not delivered by a professional who obtained a NMT certificate, but rather by a Physical Therapist trained in delivering RAC. We hypothesized that RAC would have similar short-term therapeutic effects in APDs as for patients with PD.

The classification of atypical parkinsonism includes various movement disorders, each with unique phenotypes and pathologies (25). The disorders included in this study are progressive supranuclear palsy (PSP), corticobasal syndrome (CBS), multiple system atrophy (MSA), and dementia with Lewy bodies (DLB) (25). The diagnosis of PSP can be further stratified into various subtypes, the predominant one being PSP Richardson Syndrome (PSP-RS). As the number of patients in other subcategories of PSP was too small to be grouped independently, the diagnosis of PSP was stratified into PSP-RS and PSP non-RS. There are two distinct neuropathological substrates for APDs. MSA and DLB, like idiopathic Parkinson's disease (iPD), are caused by the aggregation of α -synuclein in the brain. PSP and CBS are typically caused by tauopathies (TAU): the aggregation of 4-repeat tau in the brain (25).

In summary while there are studies describing the clinical benefits of these interventions in PD, they have largely remained unexplored in atypical parkinsonian disorders (APD), which involve early gait and balance impairment and a more rapid progression to disability and death. APD currently lack therapeutic options to target gait and balance, and based on the PD literature, music-based interventions for these disorders warrant exploration.

## Materials and methods

### Participants

Participants were recruited for this study from the Johns Hopkins Atypical Parkinsonism Center's multidisciplinary clinic between 2015 and 2019. This study included participants who had a diagnosis of PSP (26), CBS (27), MSA (28), or DLB (29) based on their respective published clinical diagnostic criteria. For participants recruited prior to the most recent PSP, DLB and MSA criteria publication (26, 29), retrospective review of clinical records was performed and applied retroactively. Autopsy confirmation of diagnosis was obtained for 3 clinically diagnosed PSP participants to date. Further inclusion criteria included slowed cadence or reduced gait velocity during physical therapy evaluations, and an ability to walk in a safe manner for at least 2 min at a time. Fifty-two patients received RAC; three participants were excluded due to incomplete testing and three participants whose diagnosis was revised to iPD were removed, resulting in 46 participants with a complete data set. Of the 46 patients analyzed, 17 were diagnosed with PSP-RS, eight with PSP-non-RS, nine with CBS, eight with MSA, and four with DLB. Demographic data is shown in Table 1. Thirty-four participants (PSP + CBS) had suspected tauopathy (TAU) [meeting probable 4-repeat tauopathy criteria (26)] and 12 had suspected alpha-synucleinopathy (SYN) (Table 1).

**Table 1:** Participants' demographic data.

	**All**	**PSP**	**PSP-RS**	**PSP-nonRS**	**CBS**	**MSA**	**DLB**
*N*	46	25	17	8	9	8	4
Male	27	15	8	7	5	4	3
Female	19	10	9	1	4	4	1
Age: mean (SD)	70.17 (7.15)	71.96 (6.61)	70.47 (6.25)	75.13 (6.62)	70.33 (5.61)	64.5 (6.12)	70 (11.52)
Disease Duration (years): mean (SD)	4.45 (2.31)	4.64 (2.17)	4.06 (1.06)	5.88 (3.31)	4.72 (3.44)	3.5 (0.76)	5.5 (2.38)
MoCA scores	20.56 (5.52)	20.79* (5.48)	20.38 (5.61)	19 (5.58)	21.56 (5.01)	24.25 (3.46)	14.75 (5.44)

*One PSP-RS participant's MoCA scores were not collected; MoCA, montreal cognitive assessment.

Written informed consent was obtained prior to participation in the study. The study protocol was approved by the Institutional Review Board at the Johns Hopkins Medical Institutions (Baltimore, MD; IRB#00062534).

### Study design

Participants were instructed to walk at their preferred pace for 2 min without any rhythmic auditory cues to collect baseline gait parameters [cadence (steps/minute), gait velocity (meters/minute), and stride length (centimeters)]. Stride length was defined as the distance between successive points of initial contact of the same foot for two consecutive steps (30). The participants then walked the same path for another 2 min with RAC and were instructed to align their steps in time with the rhythm. RAC was administered *via* a free metronome phone application (Metronome for iPhone, MetroTimer), which was set to a tempo 10% faster than the baseline cadence of each participant. After a 10–15-min break, participants walked the same path for another 2 min without RAC. The physical therapist carefully counted the number of steps for participants with research assistants who timed the trials using a smart phone timer and served as a second step counter. The path walked was identical for all participants and its length, together with the number of times that length was covered by participants, was used to calculate stride length and gait velocity [stride length = (distance traveled/number of steps)^*^2; gait velocity = distance traveled/2 min]. Gait parameters were measured at baseline, during RAC, and post-RAC. All RAC interventions were performed by the physical therapists who were part of the study team (RP, S-KH, AM) during Multi-Disciplinary Atypical Parkinsonism Clinic visits at the Johns Hopkins Atypical Parkinsonism Center. Each participant was exposed to the same indoor clinic environment and walked unaided (but closely supervised by the Physical Therapists) along the same walking path at baseline, during, and after RAC. Participants wore comfortable clothing and shoes and walked along a flat, well-lit unobstructed surface. Every effort was made to minimize outside noise. All of the above parameters were kept constant for all three walking trials.

### Statistical analysis

Shapiro-Wilk tests and Levene tests were performed to test for normal distributions and equal variances, respectively, among all data groups. Non-parametric statistical tests were applied to non-normal data or data with unequal variance, all statistical analyses to ensure adequate stringency: The Wilcoxon test and the Mann-Whitney U test. Statistical significance was set to *p* < 0.05 for all analyses.

The effects of RAC on the cadence, stride length, and gait velocity for the entire cohort and each subgroup were determined using the Wilcoxon test, to assess gait parameter change between baseline (pre-RAC) vs. during RAC, pre-RAC vs. post-RAC, and during-RAC vs. post-RAC. The Mann-Whitney U test was utilized to assess differences between subgroups in each gait parameter. A priori analyses were performed using the entire subject cohort, while subgroup analyses were considered exploratory because of unequal subgroup size and lack of power to detect statistically significant differences after correcting for multiple comparisons. Statistical analysis was performed in Python (version 3.9).

Correction for multiple comparisons was performed, setting a False Discovery Rate (FDR) by applying the Benjamini-Hochberg method [*p*-value^*^(total number of hypotheses tested)/(rank of the p-value)] (31) (full cohort analysis: 9 *p*-value samples; exploratory subgroup analyses: 54 *p*-value samples; exploratory TAU vs. SYN analyses: 27 *p*-value samples). To obtain the effect size for each comparison, Cohen's d was calculated by dividing the difference between two groups' means by their pooled standard deviation. The sample size necessary to achieve a power of 0.8 was obtained by performing power calculations using Cohen's *d* value for each comparison and an alpha level of 0.05 (using G^*^Power, version 3.1.9.7). For our subgroup analyses, we report *p*-values before correction due to the small and unequal numbers of participants in subgroups (PSP, CBS, MSA, and DLB) and the exploratory nature of these analyses. All corrected *p*-values are reported in Supplementary Table 1.

## Results

### Cohort

Participants included 27 males and 19 females, with mean (standard deviation) age of 70.17 (7.15), disease duration 4.45 (2.31) years, and total MoCA scores of 20.56 (5.52) (see Table 1). Of note, participants with DLB had lower MoCA scores than other participants [mean (SD) 14.75 (5.44) vs. 21.12 (5.25) for remaining participants]. However, MoCA scores did not have any significant associations with any gait parameters for the overall APD cohort or the subgroups (see Supplementary materials).

### Cadence

**Figure 1:**
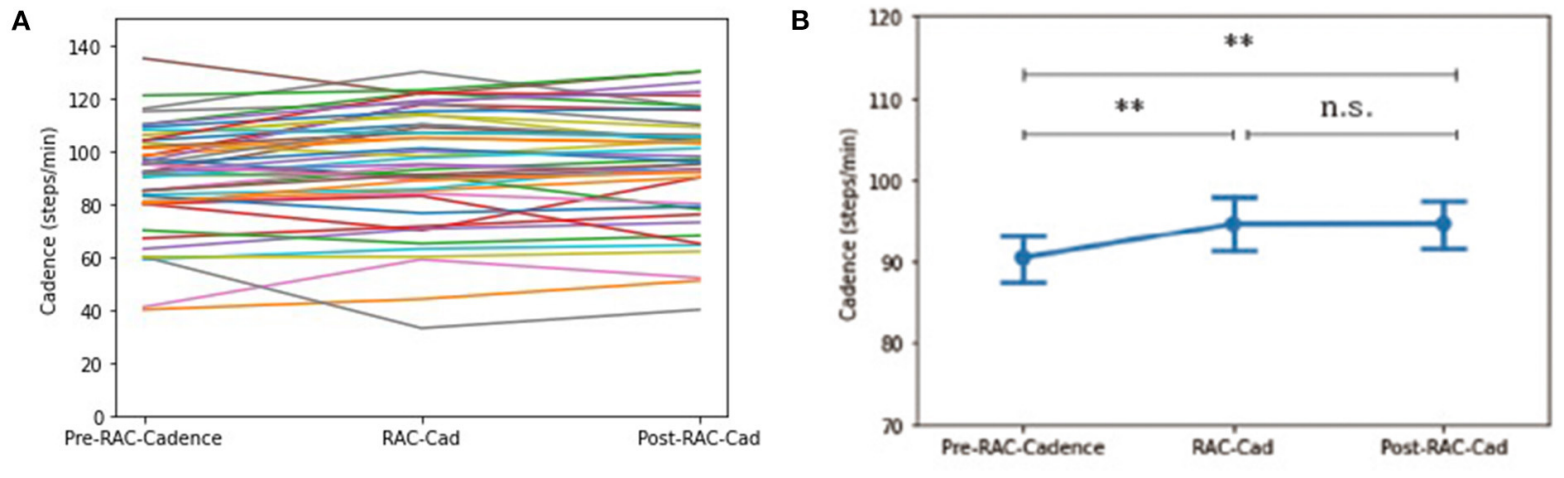
Effects of RAC on cadence in all participants. **p < 0.01; n.s. = non-significant; **(A)** spaghetti plot; **(B)** line plot with SEM (standard error of the mean); Cad, cadence; Pre, baseline.

There was a significant improvement in cadence (steps/min) for all participants when comparing baseline [90.40 (19.66)] and during-RAC [94.68 (22.23)], *p* = 0.001 (corr. *p* =0.009). This improvement was retained post-RAC [94.73 (21.12)], *p* = 0.002 (corr. *p* = 0.009) (Figure 1).

Exploratory subgroup analyses demonstrated no significant changes in cadence between subgroups at pre-RAC, during RAC, and post-RAC. A significant increase in cadence was shown in participants with PSP between baseline [87.72 (20.27)] vs. during RAC [93.98 (21.80)], *p* = 0.003; baseline vs. post-RAC [93.72 (19.18), *p* = 0.003]. In PSP, PSP-RS participants also showed significant differences between baseline (83.82 (21.58) vs. during RAC [90.03 (22.96)], *p* = 0.017 as well as baseline vs. post-RAC [89.15 (19.55)], *p* = 0.021. A trend toward improvement in cadence was seen in PSP-non-RS between baseline [96.00 (15.17)] and during RAC [102.38 (17.48)], *p* = 0.050 and baseline vs. post-RAC [103.44 (15.16)], *p* = 0.050. CBS participants also showed a significant improvement in cadence between baseline [94.00 (18.83)] and post- RAC [101.33 (21.96)], *p* = 0.039 and trend-level improvement from baseline to during RAC [99.72 (22.01)], *p* = 0.068. There was no significant cadence improvement for MSA or DLB groups (Table 2).

**Table 2:** Summary of cadence in all participants and subgroups.

		**Timeline**	**Mean (SD)**	**Median**	**SEM**	**Range**	**Comparison**	***p*-value**
Cadence (steps/min)	All (*n* = 46)	Baseline	90.40 (19.66)	93.50	2.90	40.00–135.00	Pre - During	0.001**(corr.0.009)
		During-RAC	94.68 (22.23)	96.25	3.28	33.00–130.00	Pre - Post	0.002**(corr.0.009)
		Post-RAC	94.73 (21.12)	96.50	3.11	40.00–130.00	During - Post	0.747
	PSP (*n* = 25)	Baseline	87.72 (20.27)	92.00	4.05	40.00–116.00	Pre - During	0.003**
		During-RAC	93.98 (21.80)	94.50	4.36	44.00–130.00	Pre - Post	0.003**
		Post-RAC	93.72 (19.18)	96.00	3.84	51.00–126.00	During - Post	0.833
	PSP-RS (*n* = 17)	Baseline	83.82 (21.58)	91.00	5.23	40.00–116.00	Pre - During	0.017*
		During-RAC	90.03 (22.96)	91.00	5.57	44.00–130.00	Pre - Post	0.021*
		Post-RAC	89.15 (19.55)	93.00	4.74	51.00–116.50	During - Post	0.640
	PSP-nonRS (*n* = 8)	Baseline	96.00 (15.17)	97.25	5.36	67.00–115.00	Pre - During	0.050
		During-RAC	102.38 (17.48)	103.00	6.18	71.50–122.00	Pre - Post	0.050
		Post-RAC	103.44 (15.16)	103.50	5.36	76.00–126.00	During - Post	0.623
	CBS (*n* = 9)	Baseline	94.00 (18.83)	95.00	6.28	60.00–121.00	Pre - During	0.068
		During-RAC	99.72 (22.01)	107.00	3.23	60.00–123.00	Pre - Post	0.039*
		Post-RAC	101.33(21.97)	105.00	7.32	62.00–130.00	During - Post	0.203
	MSA (*n* = 8)	Baseline	89.69 (18.01)	95.50	6.37	60.00–109.00	Pre - During	0.383
		During-RAC	90.00 (28.43)	101.25	10.05	33.00–115.00	Pre - Post	0.352
		Post-RAC	90.38 (25.82)	102.00	9.13	40.00–116.00	During - Post	0.945
	DLB (*n* = 4)	Baseline	100.50 (23.90)	93.50	11.95	80.00–135.00	Pre - During	0.625
		During-RAC	97.13 (16.97)	92.00	8.49	83.00–121.50	Pre - Post	0.109
		Post-RAC	94.88 (26.71)	92.25	13.35	65.00–130.00	During - Post	1

*p < 0.05, **p < 0.01; Significant *p*-values after FDR corrections are indicated in Bold; SD, standard deviation; SEM, standard error of the mean; corr., corrected; Pre indicates baseline.

### Velocity

**Figure 2:**
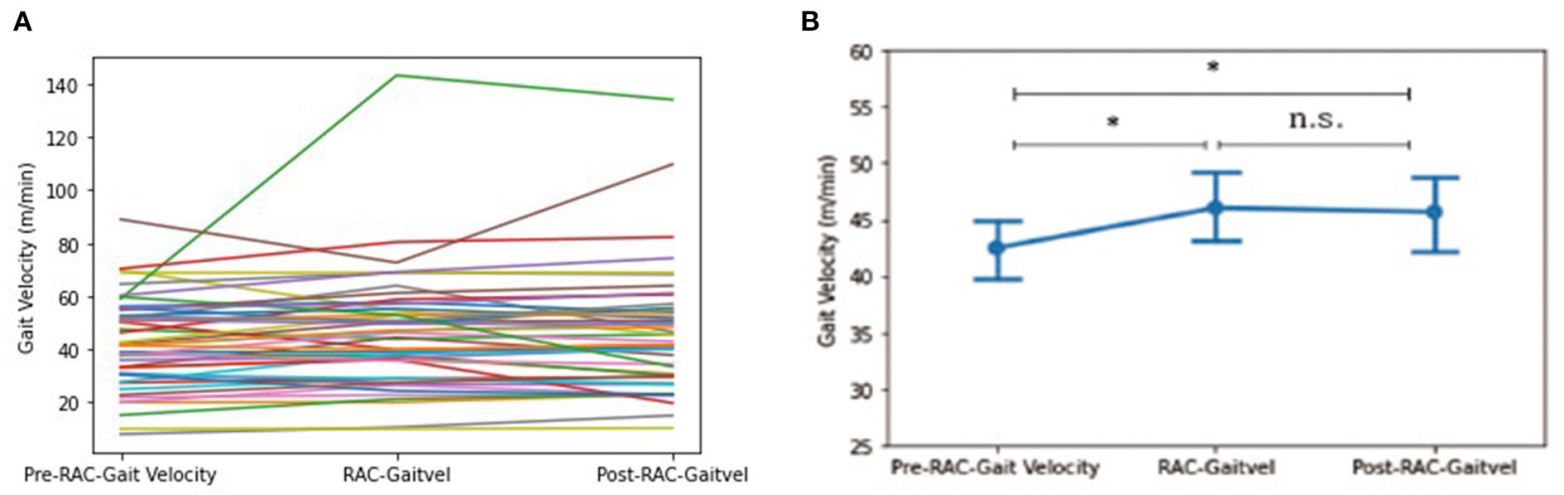
Effects of RAC on velocity in all participants. *p < 0.05; n.s., not significant; **(A)** spaghetti plot; **(B)** line plot with SEM (standard error of the mean); Gaitvel, gait velocity; m/min, meters/minute; Pre, baseline.

A significant improvement in gait velocity (m/min) was seen in all participants between baseline [Mean (standard deviation) = 42.49 (17.19)] and with-RAC [46.05 (21.96)], *p* = 0.024. This improvement was also retained during retesting 15 min later post-RAC [45.69 (23.28)], *p* = 0.039 (Figure 2).

In our exploratory analyses, between PSP-RS and PSP-nonRS, there was a trend difference (*p* = 0.051) for baseline gait velocity and a significant difference in gait velocity for during-RAC and post-RAC (*p* = 0.017, *p* = 0.007, respectively). There was a significant improvement in gait velocity in PSP participants between baseline [43.70 (14.26)] vs. during RAC [49.66 (24.00)], *p* = 0.022. PSP-nonRS showed significant improvement in velocity between both baseline [51.97 (14.05)] vs. during-RAC [66.05 (34.22)], *p* = 0.018 and baseline vs. post-RAC [65.65 (31.20)], *p* = 0.028. MSA participants also demonstrated improvement in gait velocity between both baseline [34.59 (18.74)] vs. post-RAC [41.05 (18.57)], *p* = 0.018, as well as between during RAC [38.79 (18.43)]vs. post-RAC, *p* = 0.023. There was a trend of improvement between baseline vs. during-RAC, *p* = 0.055 (Table 3).

**Table 3:** Summary of velocity in all participants and subgroups.

		**Timeline**	**Mean (SD)**	**Median**	**SEM**	**Range**	**Comparison**	***p*-value**
Gait velocity (m/min)	All (*n* = 46)	Baseline	42.49 (17.19)	41.45	2.53	8.08–89.00	Pre - During	0.024*
		During-RAC	46.05 (21.96)	45.42	3.24	10.06–143.26	Pre - Post	0.039*
		Post-RAC	45.69 (23.28)	42.37	3.43	10.36–134.11	During - Post	0.981
	PSP (*n* = 25)	Baseline	43.70 (14.27)	41.45	2.85	20.12–69.80	Pre - During	0.022*
		During-RAC	49.66 (24.00)	46.33	4.80	20.12–143.26	Pre - Post	0.141
		Post-RAC	47.73 (22.86)	45.57	4.57	22.86–134.11	During - Post	0.117
	PSP-RS (*n*= 17)	Baseline	39.80 (12.99)	37.80	3.15	20.12–69.80	Pre - During	0.221
		During-RAC	41.95 (12.44)	39.93	3.02	20.12–64.01	Pre - Post	0.890
		Post-RAC	39.31 (11.07)	40.84	2.68	22.86–60.66	During - Post	0.065
	PSP-nonRS (*n* = 8)	Baseline	51.97 (14.05)	55.93	4.97	27.43–68.58	Pre - During	0.018*
		During-RAC	66.05 (34.22)	61.95	12.10	28.96–143.26	Pre - Post	0.028*
		Post-RAC	65.65 (31.20)	60.05	11.03	29.57–134.11	During - Post	0.933
	CBS (*n* = 9)	Baseline	41.48 (19.56)	41.45	6.52	10.06–70.41	Pre - During	0.778
		During-RAC	41.36 (21.28)	39.93	7.09	10.06–80.47	Pre - Post	0.734
		Post-RAC	40.68 (22.40)	33.53	7.47	210.36–82.30	During - Post	0.496
	MSA (*n* = 8)	Baseline	34.59 (18.74)	35.20	6.63	8.08–55.63	Pre - During	0.055
		During-RAC	38.79 (18.43)	44.20	5.52	10.67–61.26	Pre - Post	0.018*
		Post-RAC	41.05 (18.57)	46.56	6.57	15.09–64.01	During - Post	0.023*
	DLB (*n* = 4)	Baseline	53.00 (25.15)	44.81	12.57	33.38–89.00	Pre - During	0.625
		During-RAC	48.54 (17.37)	42.90	8.69	35.66–72.69	Pre - Post	0.875
		Post-RAC	53.42 (39.47)	42.06	19.74	19.81–109.73	During - Post	1

*p < 0.05; SD, standard deviation; SEM, standard error of the mean; corr., corrected; Pre indicates baseline.

### Stride length

**Figure 3:**
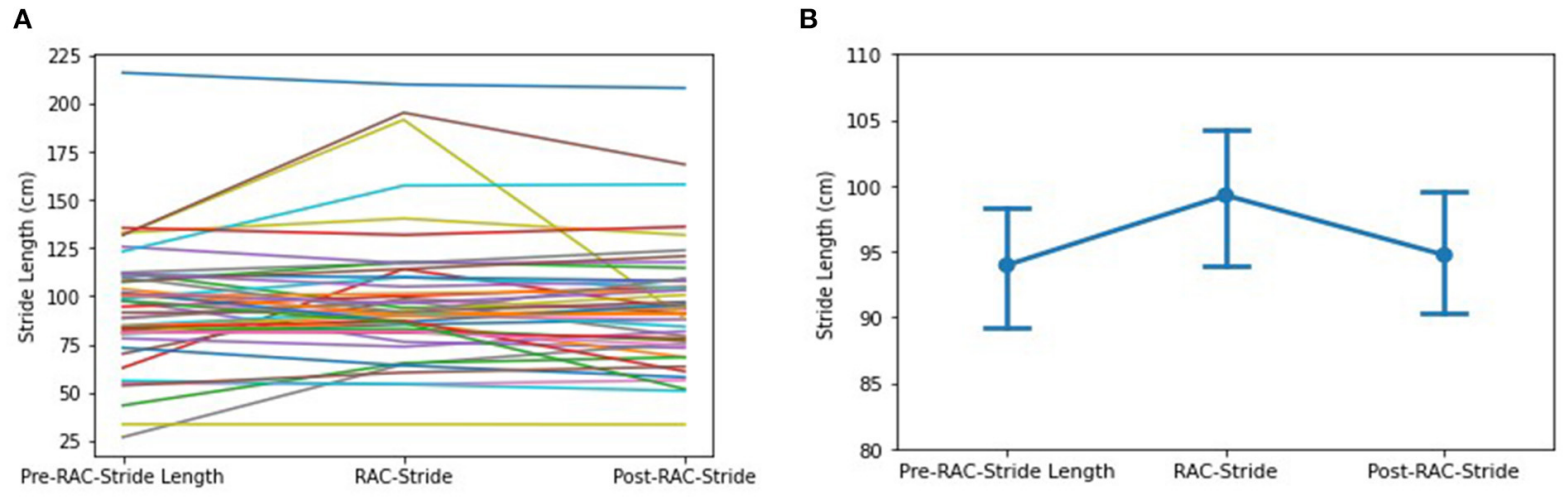
Effects of RAC on stride length in all participants. **(A)** spaghetti plot; **(B)** line plot with SEM (standard error of the mean); Stride, Stride Length; cm, centimeters; Pre, baseline.

No significant improvement in stride length (cm) was seen in the assessment of the entire cohort (Figure 3). However, subgroup exploratory analysis of PSP-nonRS and CBS showed a significant difference of stride length in during-RAC and post-RAC (*p* = 0.034, *p* = 0.049, respectively). A significant difference between PSP-RS and PSP-nonRS in post-RAC, *p* = 0.013 was also observed.

In our exploratory analyses, diagnostic subgroup analyses of PSP participants showed a significant decrease between during-RAC [106.19 (32.34)] and post-RAC [98.97 (27.64)], *p* = 0.039. MSA demonstrated a significant improvement in their stride length between baseline [79.78 (34.38)] and post-RAC [97.27(32.19)], *p* = 0.039 as well as between during RAC [91.97(33.28)] vs. post-RAC, *p* = 0.008. PSP-RS and CBS participants showed a decreasing trend (PSP-RS: during RAC [104.28(37.56)] vs. post-RAC [94.12 (30.70)], *p* = 0.057; CBS: baseline [84.87 (32.63)] vs. during-RAC [78.57 (28.81)], *p* = 0.050 (Table 4).

**Table 4:** Summary of stride length in all participants and subgroups.

		**Timeline**	**Mean (SD)**	**Median**	**SEM**	**Range**	**Comparison**	***p*-value**
Stride Length (cm)	All (*n* = 46)	Baseline	93.96 (31.22)	96.01	4.60	26.82–215.80	Pre - During	0.232
		During-RAC	99.27 (34.84)	92.05	5.14	33.53–209.70	Pre - Post	0.926
		Post-RAC	94.73 (31.64)	92.23	4.67	33.41–207.87	During - Post	0.331
	PSP (*n* = 25)	Baseline	100.49 (30.00)	94.49	6.00	62.79–215.80	Pre - During	0.179
		During-RAC	106.19 (32.34)	97.54	6.47	73.76–209.70	Pre - Post	0.672
		Post-RAC	98.97 (27.64)	93.27	5.53	27.64–207.87	During - Post	0.039*
	PSP-RS (*n* = 17)	Baseline	97.57 (34.33)	89.00	8.33	62.79–215.80	Pre - During	0.379
		During-RAC	104.28 (37.56)	92.05	9.11	73.76–209.70	Pre - Post	0.353
		Post-RAC	94.12 (30.70)	88.39	7.45	68.28–207.87	During - Post	0.057
	PSP-nonRS (*n* = 8)	Baseline	106.68 (18.08)	109.12	6.39	81.69–132.89	Pre - During	0.250
		During-RAC	110.26 (18.21)	113.39	6.44	81.08–140.21	Pre - Post	0.461
		Post-RAC	108.97 (17.11)	111.25	6.05	78.03–131.67	During - Post	0.547
	CBS (*n* = 9)	Baseline	84.87 (32.63)	97.54	10.88	33.53–135.33	Pre - During	0.050
		During-RAC	78.57 (28.81)	85.95	9.60	33.53–131.67	Pre - Post	0.250
		Post-RAC	77.37 (33.80)	63.40	11.27	33.41–135.94	During - Post	0.820
	MSA (*n* = 8)	Baseline	79.78 (34.48)	90.22	12.19	26.82–123.14	Pre - During	0.209
		During-RAC	91.97 (33.28)	89.61	11.76	54.25–157.28	Pre - Post	0.039*
		Post-RAC	97.27 (32.19)	98.15	11.40	56.39–157.89	During - Post	0.008**
	DLB (*n* = 4)	Baseline	101.96 (24.16)	97.54	12.08	81.08–131.67	Pre - During	0.625
		During-RAC	117.20 (52.85)	96.01	26.43	81.69–195.07	Pre - Post	0.875
		Post-RAC	102.87 (47.85)	91.14	23.92	60.96–168.25	During - Post	0.250

*p < 0.05, **p < 0.01; Significant *p*-values after FDR corrections are indicated in Bold; SD, standard deviation; SEM, standard error of the mean; corr., corrected; Pre indicates base.

### Suspected TAU and SYN participants

The TAU (PSP + CBS) cohort experienced significant improvements in cadence between baseline [89.38 (19.82)] vs. during RAC [95.50 (21.67)], *p* = 0.00039 (corr. *p* = 0.004), and between baseline vs. post-RAC [95.74 (19.91)], *p* = 0.00024 (corr. *p* = 0.004). The TAU cohort also experienced significant improvements in gait velocity between baseline [43.11 (15.55)] vs. during RAC [47.46 (23.29)], *p* = 0.038. In contrast, the SYN cohort did not demonstrate significant improvements in cadence or gait velocity (in Supplementary Table 2).

Interestingly, a significant difference in cadence was seen in the delta baseline vs. during RAC [TAU: 6.11(7.92)] vs. SYN [−0.91 (10.41), *p* = 0.023], and delta baseline vs. post-RAC between TAU and SYN [TAU: 6.35 (8.17) vs. SYN (−1.42 (8.74)], *p* = 0.007. When comparing changes in stride length between baseline vs. during RAC, the SYN cohort experienced an average improvement of 13.21 (22.32) cm while the TAU cohort only experienced a 2.53 (16.81) cm increase in stride length, *p* = 0.056. When comparing the change in stride length between baseline vs. post-RAC, the TAU cohort experienced an average shortening in stride length of −3.18 (15.16) cm, while the SYN cohort experienced an average lengthening in stride length of 11.96 (21.35) cm, *p* = 0.023 (in Supplementary Table 3).

### Effect size and sample size calculations

The comparisons between baseline and during-RAC for cadence had effect size of *d* = 0.204, and power calculation revealed that a total sample size of 199 participants would be necessary to yield a power of 0.8 to detect differences at alpha of 0.05. A total sample size of 251 participants would be necessary to yield a power of 0.8 for a comparison of change in gait velocity between baseline and during-RAC (*d* = 0.182) (in Supplementary Table 4).

## Discussion

This pilot study aimed to determine whether rhythmic cues using a metronome improve gait parameters (e.g., cadence, velocity, and stride length) in patients with atypical parkinsonian disorders. Our data demonstrate a significant improvement in cadence and velocity in all the participants receiving RAC, an improvement which was retained 10–15 min following RAC intervention. In particular, cadence improvements across all participants remained statistically significant between baseline and during RAC as well as between baseline vs. post-RAC after correcting for multiple comparisons.

Prior studies have demonstrated the effectiveness of RAS in improving gait parameters in neurological movement disorders (1, 3, 4, 9–22, 24). However, the effect of RAS on quantitative gait measurements in APD accounting for diagnostic subtype and therefore suspected neuropathological substrate has not been evaluated. To date, only one open label pilot study tested a home-based music-cued therapy program in five participants with progressive supranuclear palsy (PSP). After the 4-week program (eight sessions), all five participants reported being satisfied with the therapy and some exhibited clinically (but not statistically) significant improvements in their gait velocity and stride length variability (32).

Several mechanisms have been proposed for the therapeutic effects of rhythmic cues in patients with movement disorders, particularly PD. Due to impairments of the basal ganglia and loss of frontostriatal connectivity, patients with PD experience a loss of rhythm perception and timing, resulting in various gait abnormalities in PD, including freezing of gait, decreased stride length and gait velocity, and a rhythmically unstable walking pace (3). External auditory cueing may activate cerebello-thalamic networks that are generally spared in PD and this may compensate for the PD-associated basal ganglia abnormalities (4, 33). In addition, it has been hypothesized that the beneficial effects of rhythmic cues may be partly due to modulation of brain activity in the pedunculopontine nucleus (PPN) (33, 34). Another possible mechanism for rhythmic cueing in parkinsonism involves rhythmic entrainment: motor synchronization with the auditory system, which remains relatively intact in PD (13). Schaefer (35) has summarized four possible mechanisms of the use of rhythmic cues in motor rehabilitation including (1) accelerated motor learning, (2) qualitatively different motor learning, (3) obtaining temporal skills, and (4) motivation.

APD may also benefit from rhythmic cueing *via* these mechanisms since APD and PD share overlapping affected neural pathways and clinical features, including gait slowing and freezing (36). In particular, the gait parameters of progressive supranuclear palsy (PSP) and PD were substantially similar when gait was recorded at self-selected, fast, very fast, slow, and very slow speed (37). They reported that PSP, PD, and control groups showed a strong linear relationship between cadence and stride length. This finding was replicated in our data, which showed strong correlations in all participants between cadence, velocity, and stride length at baseline, with-RAC, and post-RAC (see Supplementary Table 3).

Furthermore, we found different patterns of improvement in gait parameters for each diagnostic subgroup. It is worth noting that while the group that experienced the greatest improvements was PSP-RS, this was the largest subgroup (*n* = 17). It is therefore possible that differences in improvement between diagnostic subgroups may be a function of low sample size and requires validation in a future prospective study. For example, DLB, which is the smallest group (*n* = 4), showed less response to RAC. The degree of within-diagnostic-group variance was also high, also potentially contributing to the difference in response to RAC between subgroups. The standard deviation and standard error of the mean of DLB were larger than those of other groups, such as PSP-RS. Finally, multitasking difficulty in patients with DLB (following directions, rhythmic cues while walking) due to more severe cognitive dysfunction than in other groups may also contribute to differences in response to RAC.

Substantial clinical and pathological heterogeneity in APD still impedes early diagnosis of APD (38). It will be essential to understand the neural mechanisms of rhythmic entrainment in APD using neuroimaging techniques, such as EEG, MEG, functional near-infrared spectroscopy, or functional MRI. From a therapeutic perspective, investigating neural mechanisms of RAC in APD may allow us to determine the effectiveness of rhythmic cueing on individual patients, and allow for a precision medicine approach to tailoring therapeutic intervention based on diagnostic subgroup.

Several limitations of this study should be considered. Though clinical diagnostic criteria were stringently applied, autopsy confirmation of clinical diagnosis was not available for much of the cohort. A larger sample size is required to validate the efficacy of RAC in APD patients. Larger diagnostic subgroup samples would enable us to confirm differences in responses to RAC and conduct cluster analyses to determine unique characteristic of each subgroup that may aid development of more tailored RAC interventions. Furthermore, the results of this study provide guidance concerning the effect size and calculation of sample sizes required for future controlled clinical trials of RAC in patients with APD. Until that time, we emphasize that the subgroup analyses reported here should be considered exploratory. Additionally, this study investigated only the immediate effects of rhythmic cues by giving a 10% increase over baseline cadence for all participants and long-term effects of cueing were not assessed. Use of control groups (idiopathic Parkinson's disease and healthy older individuals) and longer follow up with repeated RAC interventions in APD are warranted, as is the comparison to natural history data. Freezing of gait was not formally assessed during the study and could have impacted our outcomes in ways we did not measure. Another limitation of the study involves the possibility of learning effects across trials, since the trials occurred in close temporal proximity. A cross-over study design would be warranted to account for learning effects as an influence on participants' gait performance. We also acknowledge that despite our best efforts minor errors in step count and distance recording could have occurred, though it is doubtful that they would impact our results. A motion capture system or electronic walk system such as GAITRite^®^ can be used in the future studies to avoid potential errors in manually counting steps and distance walked. Whether RAC in APD can be effectively used in the home environment (e.g., potentially with the caregivers) and whether it can help prevent falls are key questions for future investigation. In addition, given the consideration of each APD individual's functional needs and safety level, customizing the rhythmic cues may improve response and increase intervention safety. The decision whether the patient's gait velocity (*via* cadence) needs to be accelerated or decelerated can be made jointly by physical therapists, patients, and caregivers. For a functional transition into patients' daily life, a neurologic music therapist may co-facilitate rhythmic interventions with physical therapists by adding live music, advanced gait training (e.g., walking outside on different surfaces, stop and go), and pre-gait exercises through musical patterns (i.e., patterned sensory enhancement).

The present study is the first to our knowledge to demonstrate that short-term RAC may improve relevant gait parameters in patients with APD. The use of rhythmic cues and/or music in the context of an isochronous rhythmic stimulus may be an easily accessible and effective way to improve functional gait performance in patients with APD. While our study was limited by sample heterogeneity and small subgroup sample size, it paves the way for larger trials of rhythmic cueing for patients with atypical parkinsonism.

## Data availability statement

The raw data supporting the conclusions of this article will be made available by the authors upon reasonable request, without undue reservation.

## Ethics statement

The studies involving human participants were reviewed and approved by Institutional Review Board at the Johns Hopkins Medical Institutions (Baltimore, MD; IRB#00062534). The patients/participants provided their written informed consent to participate in this study.

## Author contributions

AP: research project: conception, organization, and execution; statistical analysis: design, review, and critique; manuscript preparation: writing of the first draft, review, and critique. GD: statistical analysis: design, execution, review, and critique; manuscript preparation: writing of the first draft, review, and critique. KK: statistical analysis: execution, review, and critique; manuscript preparation: writing of the first draft, review, and critique. BT: statistical analysis: design, execution, review, and critique; manuscript preparation: review and critique. RP, S-KH, and AM: research project: conception, organization, and execution; manuscript preparation: review and critique. JB: research project: conception; statistical analysis: review and critique; manuscript preparation: review and critique. All authors contributed to the article and approved the submitted version.

## Conflict of interest

The authors declare that the research was conducted in the absence of any commercial or financial relationships that could be construed as a potential conflict of interest.

## Publisher's note

All claims expressed in this article are solely those of the authors and do not necessarily represent those of their affiliated organizations, or those of the publisher, the editors and the reviewers. Any product that may be evaluated in this article, or claim that may be made by its manufacturer, is not guaranteed or endorsed by the publisher.
