# Supplementary material for: Rhythmic auditory cueing in atypical parkinsonism: A pilot study
Source: Front Neurol. 2022 Oct 28;13:1018206. doi: 10.3389/fneur.2022.1018206 (PMC9650086; doi:10.3389/fneur.2022.1018206)
Supplement: Supplementary file 1 [file Data_Sheet_1.PDF]

Montreal Cognitive Assessment (MoCa) Scores Data

1. **All Patients (N =45) *One participant's (PSP) MoCA scores were not collected**

   A. Cadence

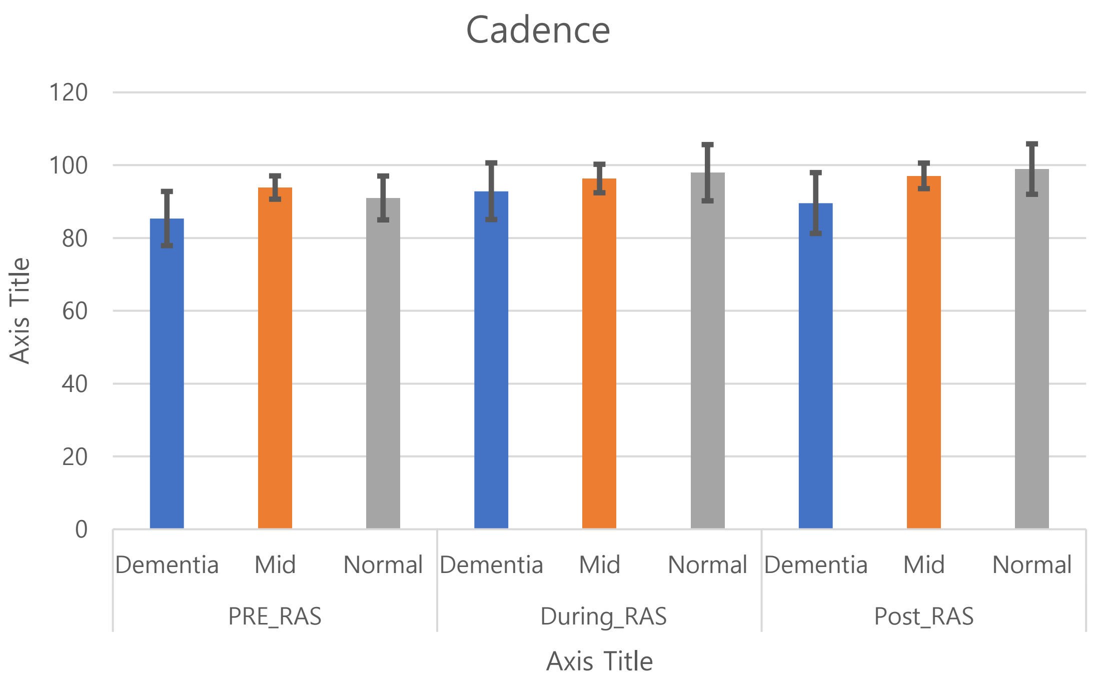

	PRE_RAS			During_RAS			Post_RAS		
	Dementia	MCI	Normal	Dementia	MCI	Normal	Dementia	MCI	Normal
Count	10	28	7	10	28	7	10	28	7
Mean	85.35	93.86	91	92.85	96.34	97.93	89.60	97.07	98.93
Std	23.53	16.94	15.93	24.61	20.67	20.42	26.43	18.57	18.34
Median	93	95	92	90	96.50	100	85.25	96.50	101
SEM	7.44	3.20	6.02	7.78	3.91	7.72	8.36	3.51	6.93

Note: Dementia (≤17); Mild Cognitive Impairment (18-25); Normal (≥26)

B. Velocity

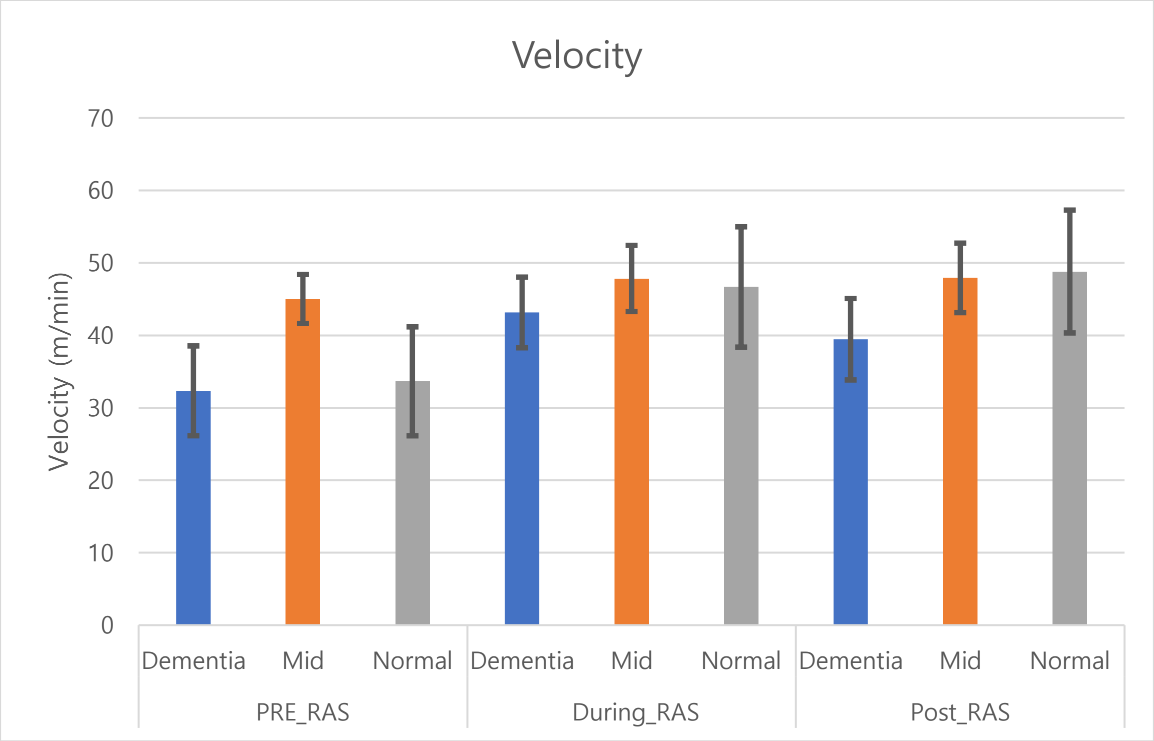

	PRE_RAS			During_RAS			Post_RAS		
	Dementia	MCI	Normal	Dementia	MCI	Normal	Dementia	MCI	Normal
Count	10	28	7	10	28	7	10	28	7
Mean	32.34	45.02	33.65	43.16	47.85	46.68	39.46	47.93	48.81
Std	17.54	17.89	21.26	15.42	24.20	21.97	17.78	25.40	22.44
Median	33.51	46.94	36.15	36.96	48.01	46.94	32.31	44.27	49.99
SEM	6.20	3.38	7.52	4.88	4.57	8.30	5.62	4.80	8.48

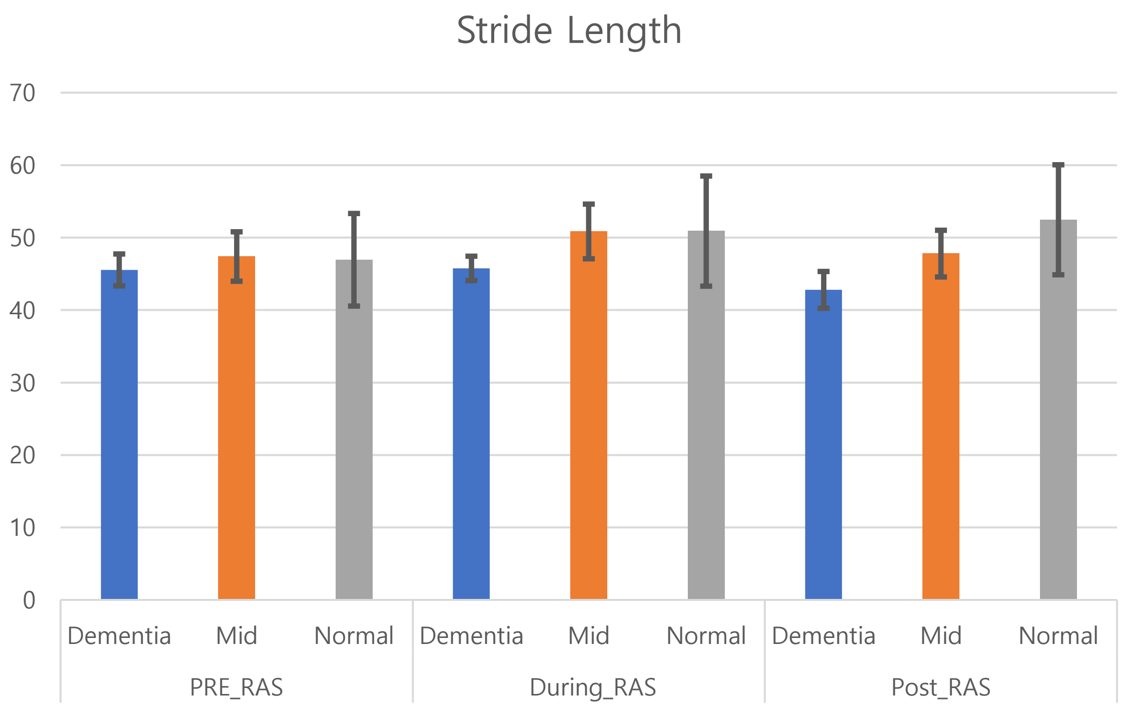

	PRE_RAS			During_RAS			Post_RAS		
	Dementia	MCI	Normal	Dementia	MCI	Normal	Dementia	MCI	Normal
Count	10	28	7	10	28	7	10	28	7
Mean	45.54	47.39	46.94	45.75	50.85	50.90	42.79	47.79	17.06
Std	6.96	18.03	16.92	5.32	19.96	20.12	8.02	17.06	20.09
Median	42.22	48.77	49.38	44.81	46.48	54.86	40.84	46.63	52.12
SEM	2.20	3.41	6.39	1.68	3.77	7.60	2.54	3.22	7.59

2. **Sub-Groups (PSP, CBS, MSA, DLB)**

A. **Cadence**

i. **Pre_RAS**

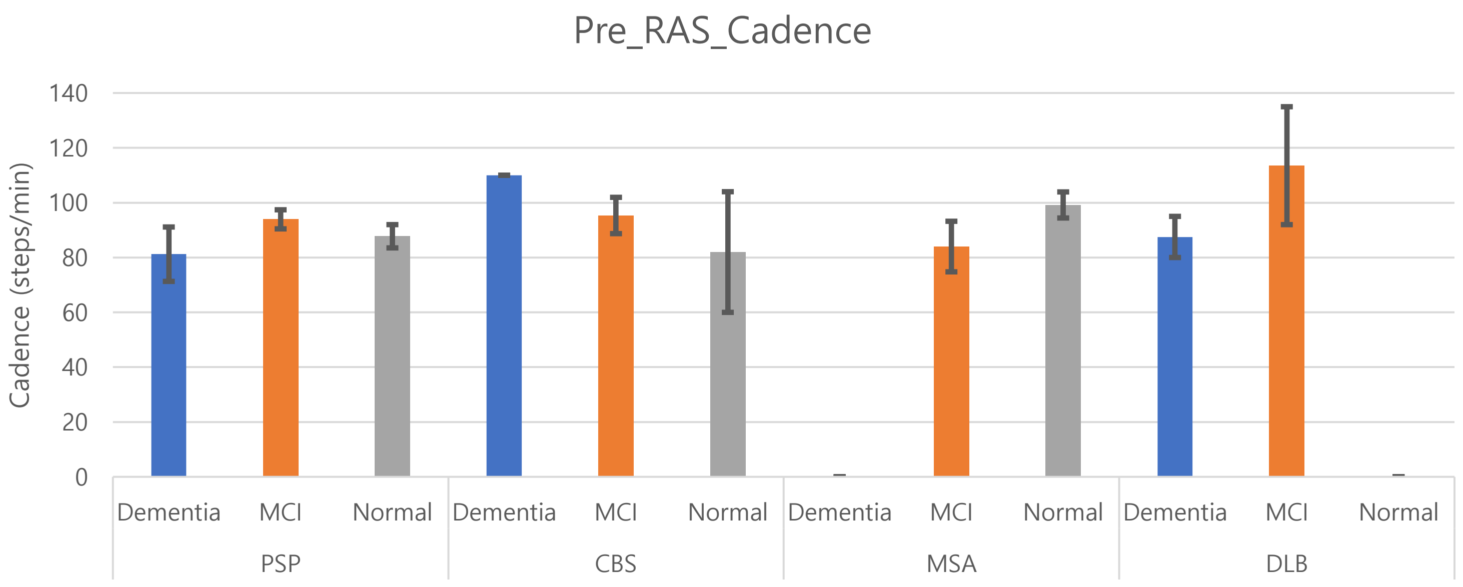

	PSP			CBS			MSA			DLB		
	Dementia	MCI	Normal	Dementia	MCI	Normal	Dementia	MCI	Normal	Dementia	MCI	Normal
N	7	15	2	1	6	2	0	5	3	2	2	n/a
Mean	81.21	93.93	87.75	110	95.33	82.00	n/a	84.00	99.17	87.50	113.50	n/a
Std	26.25	13.47	6.01	n/a	16.21	31.11	n/a	20.63	8.25	10.61	30.40	n/a
Median	91	95.00	87.75	n/a	90.00	82.00	n/a	80.00	101.50	87.50	113.50	n/a
Range	41-116	63-115	83.50-92	n/a	80-121	60-104	n/a	60-109	90-106	80-95	92-135	n/a
SEM	9.92	3.48	4.25	n/a	6.62	22.00	n/a	9.22	4.76	7.50	21.5	n/a

ii. During_RAS

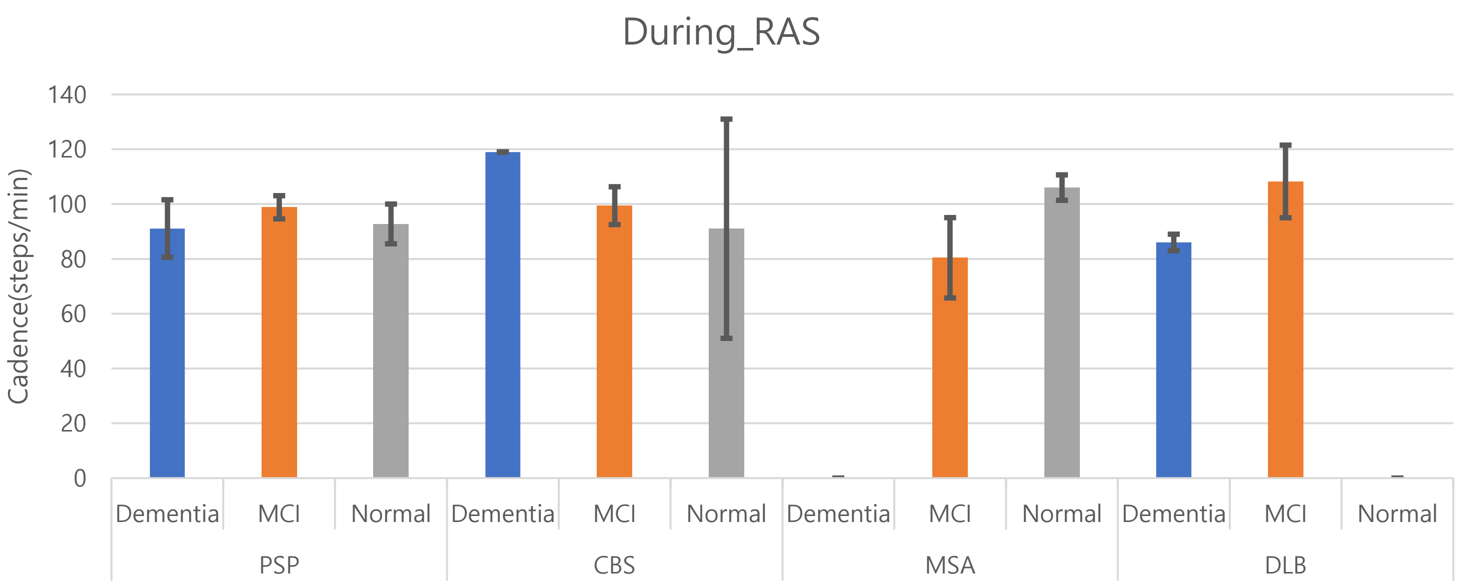

	PSP			CBS			MSA			DLB		
	Dementia	MCI	Normal	Dementia	MCI	Normal	Dementia	MCI	Normal	Dementia	MCI	Normal
N	7	15	2	1	6	2	0	5	3	2	2	n/a
Mean	88.00	98.83	96.75	122.50	101.08	91.50	n/a	81.40	105.33	78.75	111.00	n/a
Std	27.61	11.31	1.77	n/a	17.19	41.72	n/a	29.83	4.04	19.45	26.87	n/a
Median	78.00	97.00	96.75	n/a	100.00	91.50	n/a	80	106	78.75	111.00	n/a
Range	52-126	73-117	95.50-98	n/a	79-130	62-121	n/a	40-116	101-109	65.00-92.50	92-130	n/a
SEM	10.44	2.92	1.25	n/a	7.02	29.5	n/a	13.34	2.33	13.75	19.00	n/a

iii. Post_RAS

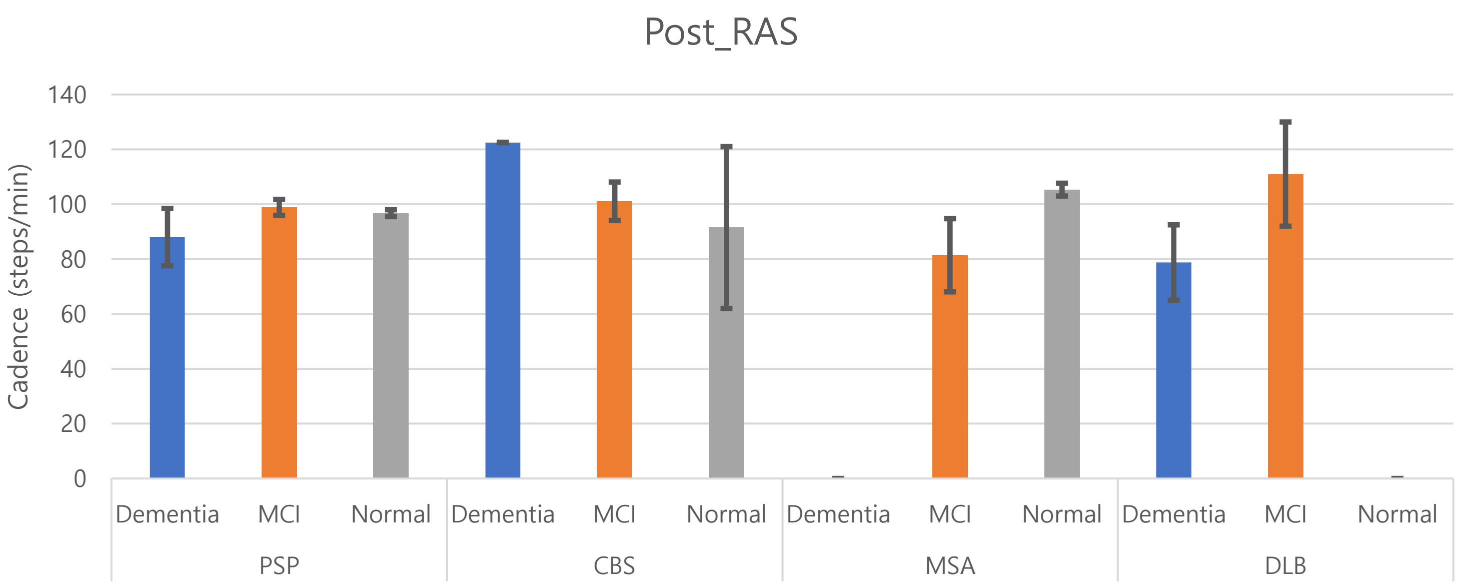

	PSP			CBS			MSA			DLB		
	Dementia	MCI	Normal	Dementia	MCI	Normal	Dementia	MCI	Normal	Dementia	MCI	Normal
N	7	15	2	1	6	2	0	5	3	2	2	0
Mean	37.62	48.77	38.71	55.63	39.54	40.23	n/a	30.30	41.76	35.89	70.10	n/a
Std	14.64	12.92	3.88	n/a	14.24	42.67	n/a	21.47	13.59	3.56	26.73	n/a
Median	37.80	47.55	38.71	n/a	35.97	40.23	n/a	21.95	42.67	35.89	70.10	n/a
Range	20.12-60.35	31.09-69.80	35.97-41.45	n/a	22.86-59.74	10.06-70.41	n/a	8.08-55.63	27.74-54.86	33.38-38.40	51.21-89.00	n/a
SEM	5.53	3.34	2.74	n/a	5.81	30.18	n/a	9.60	7.84	2.51	11.51	n/a

B. Gait Velocity

i. Pre_RAS

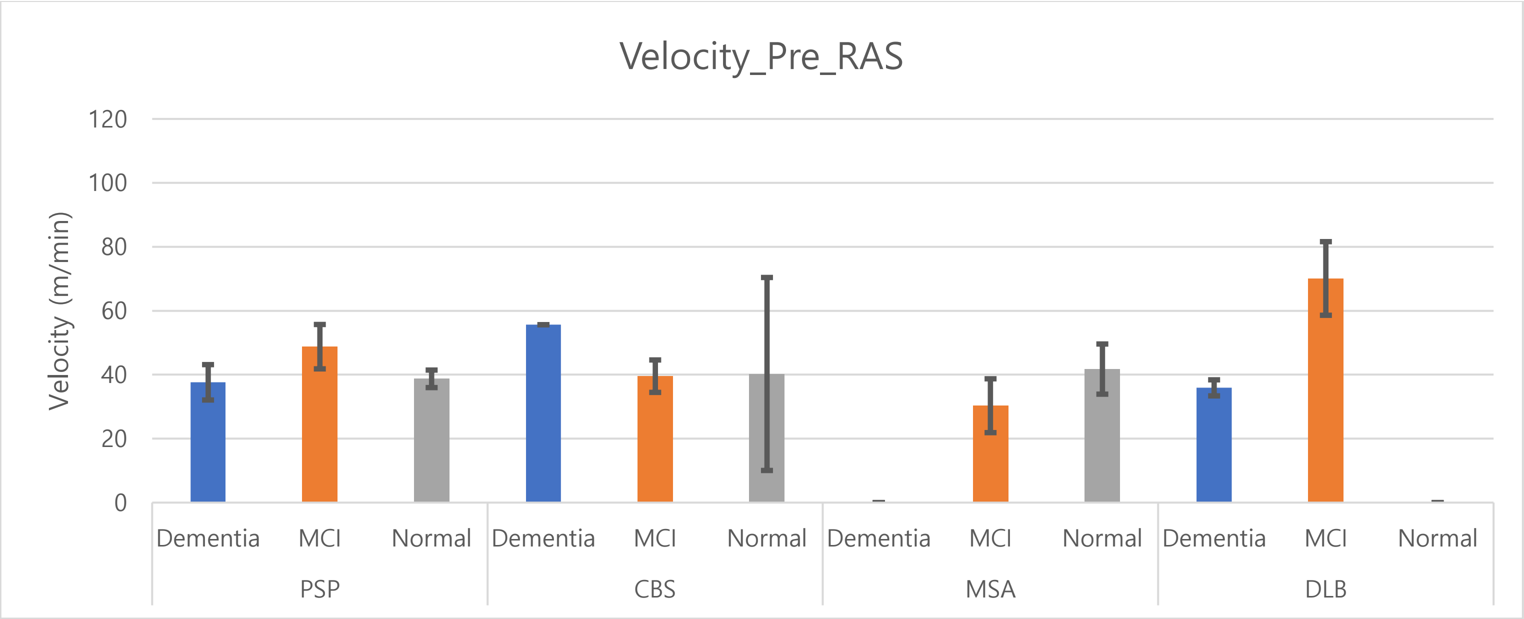

	PSP			CBS			MSA			DLB		
	Dementia	MCI	Normal	Dementia	MCI	Normal	Dementia	MCI	Normal	Dementia	MCI	Normal
N	7	15	2	1	6	2	0	5	3	2	2	0
Mean	37.62	48.77	38.71	55.63	39.54	40.23	n/a	30.30	41.76	35.89	70.10	n/a
Std	14.64	12.92	3.88	n/a	14.24	42.67	n/a	21.47	13.59	3.56	26.73	n/a
Median	37.80	47.55	38.71	n/a	35.97	40.23	n/a	21.95	42.67	35.89	70.10	n/a
Range	20.12-60.35	31.09-69.80	35.97-41.45	n/a	22.86-59.74	10.06-70.41	n/a	8.08-55.63	27.74-54.86	33.38-38.40	51.21-89.00	n/a
SEM	5.53	3.34	2.74	n/a	5.81	30.18	n/a	9.60	7.84	2.51	11.51	n/a

ii. During_RAS

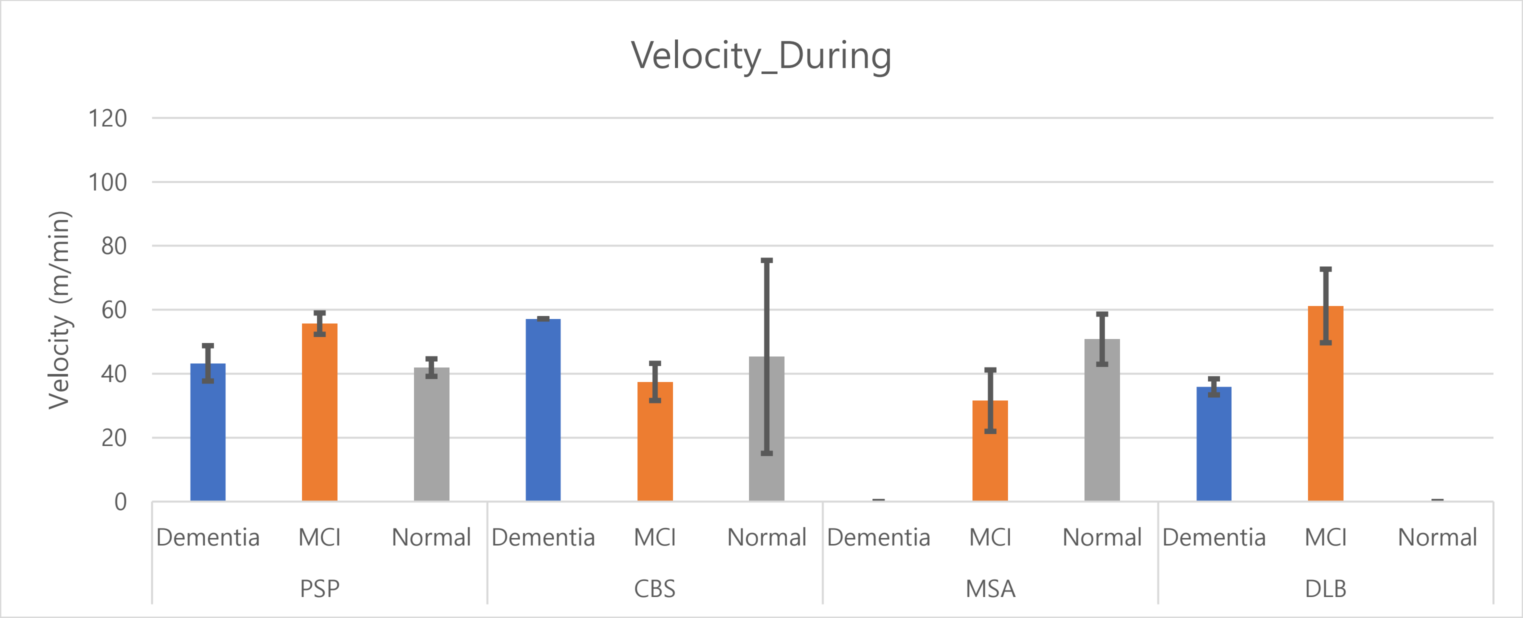

	**PSP**			**CBS**			**MSA**			**DLB**		
	Dementia	MCI	Normal	Dementia	MCI	Normal	Dementia	MCI	Normal	Dementia	MCI	Normal
N	7	15	2	1	6	2	0	5	3	2	2	0
Mean	39.89	53.39	45.11	61.19	35.38	46.33	n/a	33.92	52.93	27.13	79.71	n/a
Std	18.08	25.37	6.90	n/a	12.45	50.86	n/a	18.99	12.15	10.35	42.46	n/a
Median	30.18	45.72	45.11	n/a	31.85	46.33	n/a	23.16	54.86	27.12	79.71	n/a
Range	22.86-74.37	26.82-134.11	40.23-49.99	n/a	22.86-57.15	10.36-82.30	n/a	15.09-55.63	39.93-64.01	19.81-34.44	49.68-109.73	n/a
SEM	6.83	6.55	4.88	n/a	5.08	35.97	n/a	8.49	7.02	7.32	30.02	n/a

iii. Post_RAS

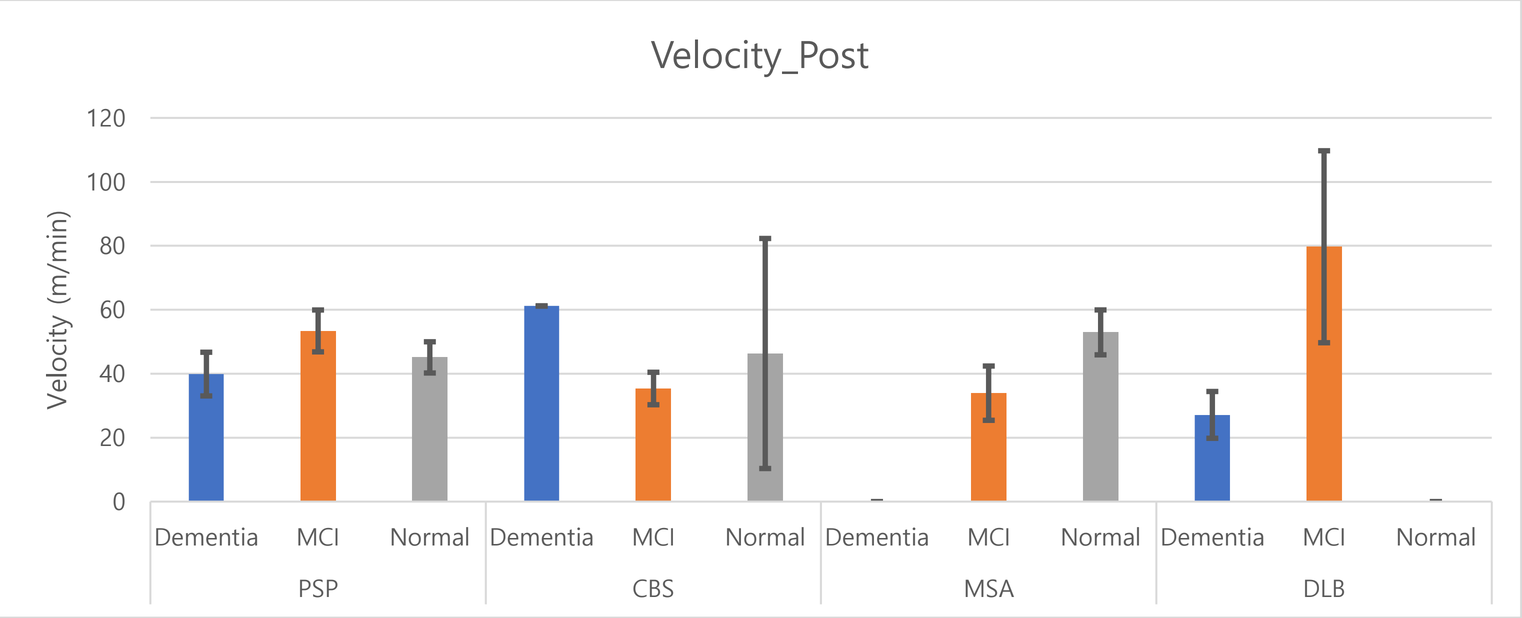

	PSP			CBS			MSA			DLB		
	Dementia	MCI	Normal	Dementia	MCI	Normal	Dementia	MCI	Normal	Dementia	MCI	Normal
N	7	15	2	1	6	2	0	5	3	2	2	0
Mean	39.89	53.39	45.11	61.19	35.38	46.33	n/a	33.92	52.93	27.13	79.71	n/a
Std	18.08	25.37	6.90	n/a	12.45	50.86	n/a	18.99	12.15	10.35	42.46	n/a
Median	30.18	45.72	45.11	n/a	31.85	46.33	n/a	23.16	54.86	27.12	79.71	n/a
Range	22.86-74.37	26.82-134.11	40.23-49.99	n/a	22.86-57.15	10.36-82.30	n/a	15.09-55.63	39.93-64.01	19.81-34.44	49.68-109.73	n/a
SEM	6.83	6.55	4.88	n/a	5.08	35.97	n/a	8.49	7.02	7.32	30.02	n/a

C. **Stride Length**

i. **Pre_RAS**

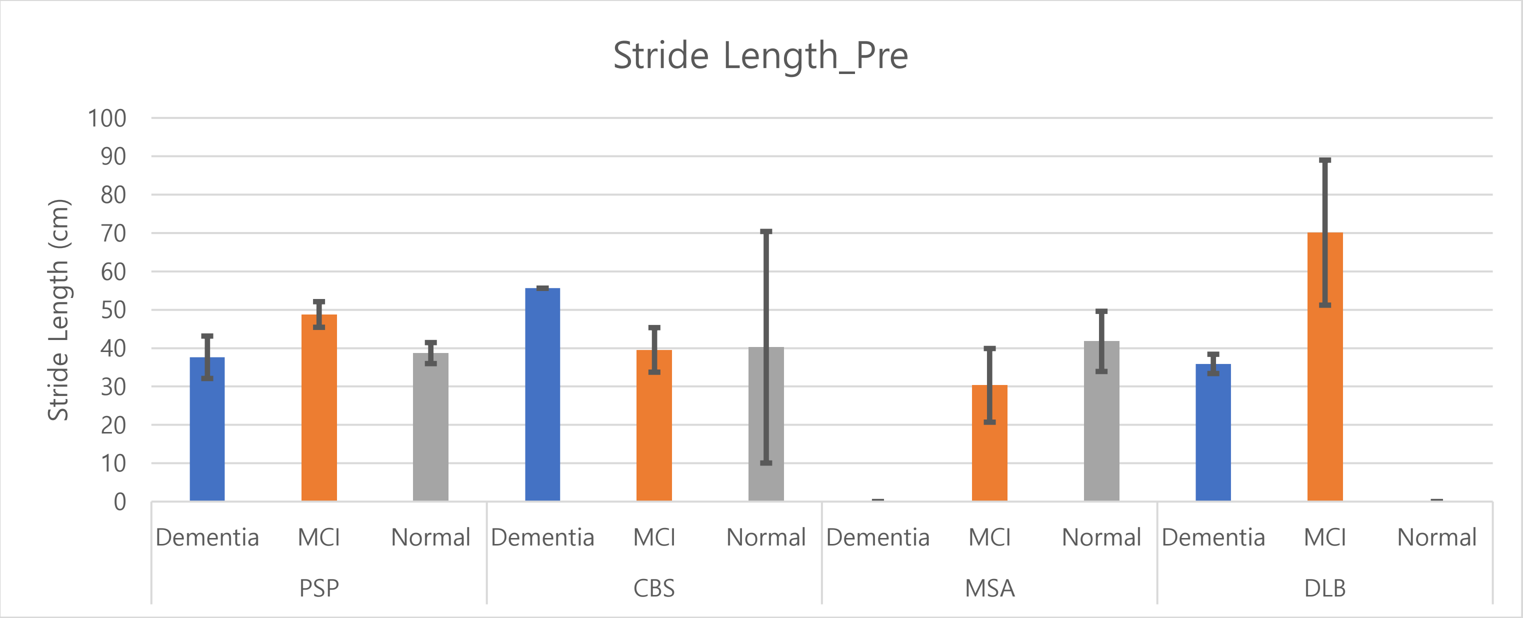

	PSP			CBS			MSA			DLB		
	Dementia	MCI	Normal	Dementia	MCI	Normal	Dementia	MCI	Normal	Dementia	MCI	Normal
N	7	15	2	1	6	2	0	5	3	2	2	0
Mean	37.62	48.77	38.71	55.63	39.54	40.23	n/a	30.30	41.76	35.89	70.10	n/a
Std	14.64	12.92	3.88	n/a	14.23	42.67	n/a	21.47	13.59	3.56	26.73	n/a
Median	37.80	47.55	38.71	n/a	35.97	40.23	n/a	21.95	42.67	35.89	70.10	n/a
Range	20.11-60.35	31.09-69.80	35.97-41.45	n/a	22.86-59.74	10.06-70.41	n/a	8.08-55.63	27.74-54.86	33.38-38.40	51.21-89.00	n/a
sem	5.53	3.34	2.74	n/a	5.81	30.18	n/a	9.60	7.84	2.51	18.90	n/a

ii. During_RAS

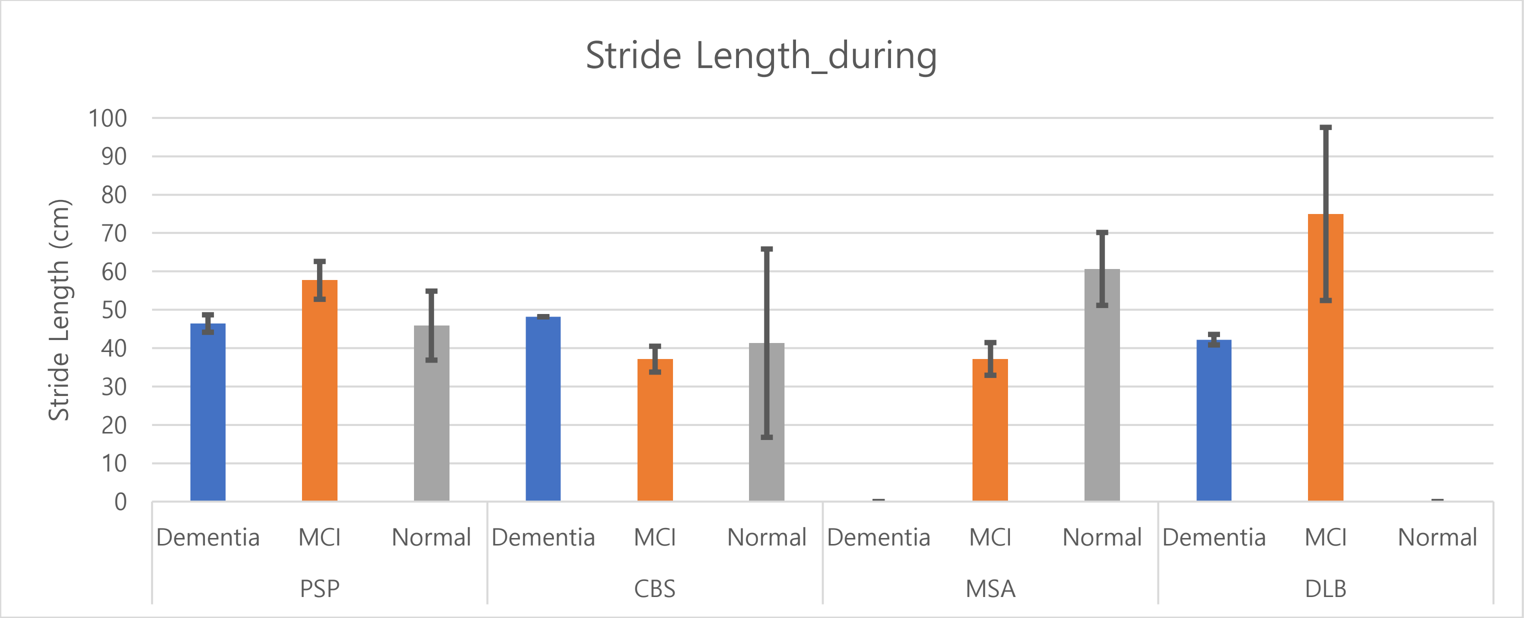

	PSP			CBS			MSA			DLB		
	Dementia	MCI	Normal	Dementia	MCI	Normal	Dementia	MCI	Normal	Dementia	MCI	Normal
N	7	15	2	1	6	2	0	5	3	2	2	0
Mean	46.42	57.67	45.87	48.16	37.13	41.30	n/a	37.19	60.65	42.21	74.98	n/a
Std	6.01	19.11	12.72	n/a	8.27	34.70	n/a	9.52	16.42	1.94	31.90	n/a
Median	44.81	49.98	45.87	n/a	37.50	41.30	n/a	32.61	57.00	42.21	74.98	n/a
Range	40.54-58.52	38.10-104.85	36.88-54.86	n/a	27.13-45.72	16.76-65.84	n/a	27.13-50.60	46.33-78.64	40-84-43.59	52.43-97.54	n/a
SEM	2.27	4.93	8.99	n/a	3.37	24.54	n/a	4.26	9.50	1.37	22.56	n/a

iii. Post_RAS

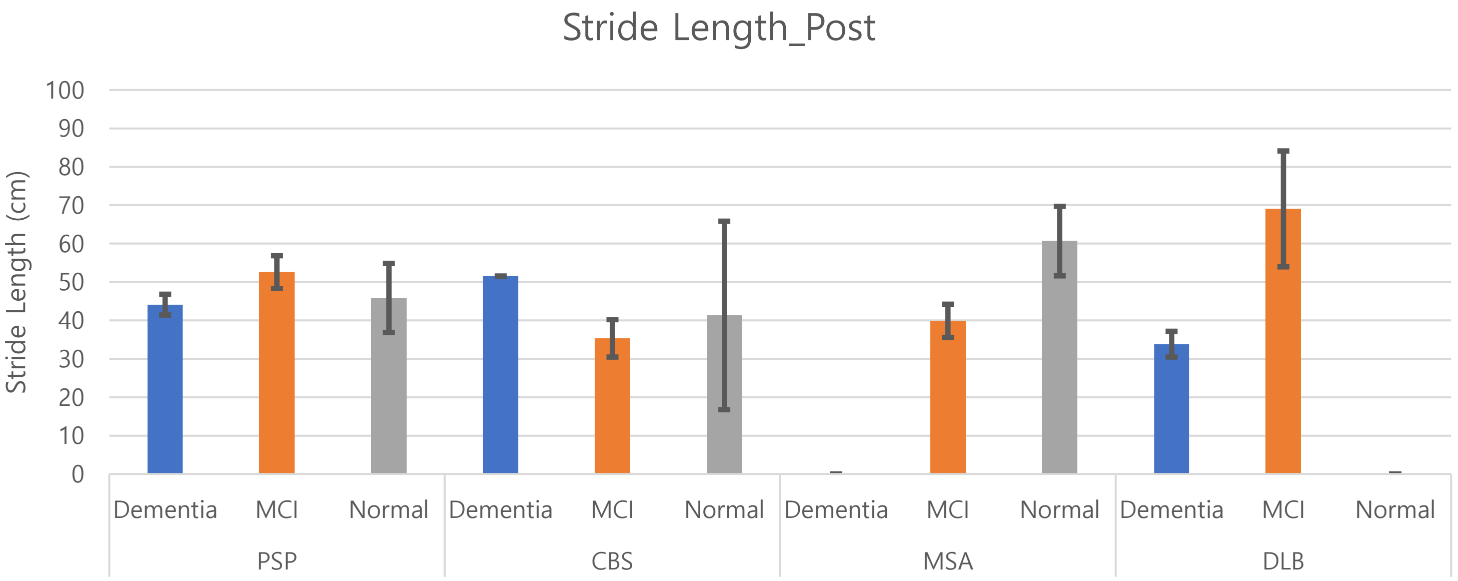

	PSP			CBS			MSA			DLB		
	Dementia	MCI	Normal	Dementia	MCI	Normal	Dementia	MCI	Normal	Dementia	MCI	Normal
N	7	15	2	1	6	2	0	5	3	2	2	0
Mean	44.11	52.58	45.87	51.51	35.33	41.30	n/a	39.90	60.66	33.82	69.04	n/a
Std	7.16	16.54	12.72	n/a	11.94	34.70	n/a	9.65	16.46	4.74	21.34	n/a
Median	42.06	47.24	45.87	n/a	30.33	41.30	n/a	37.80	57.00	33.83	69.04	n/a
Range	38.40-58.83	34.14-103.94	36.88-54.86	n/a	25.42-54.56	16.76-65.84	n/a	28.19-51.51	46.33-78.64	30.48-37.19	53.95-84.12	n/a
SEM	2.71	4.27	8.99	n/a	4.87	24.54	n/a	4.32	9.05	3.35	15.09	n/a

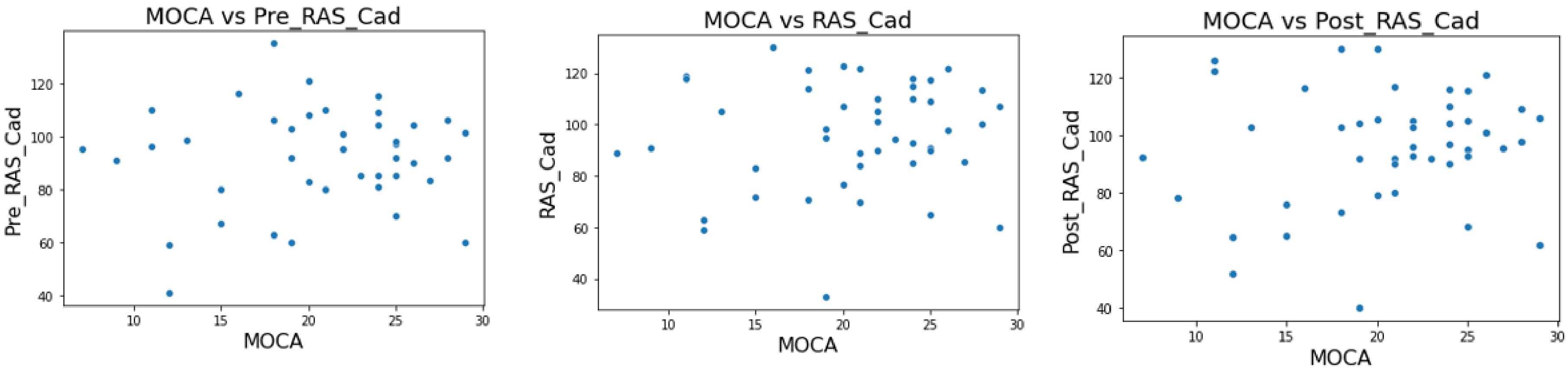

SpearmanrResult(correlation=nan, pvalue=nan) SpearmanrResult(correlation=nan, pvalue=nan) SpearmanrResult(correlation=nan, pvalue=nan)

Figure 1. Associations between MoCa scores and Cadence (steps/min) in Pre, During, and Post RAS (All patients cohort, n=45)

Note: One PSP-RS participant's MoCA scores were not collected; MoCA: Montreal Cognitive Assessment; Cad = cadence; Pre = baseline

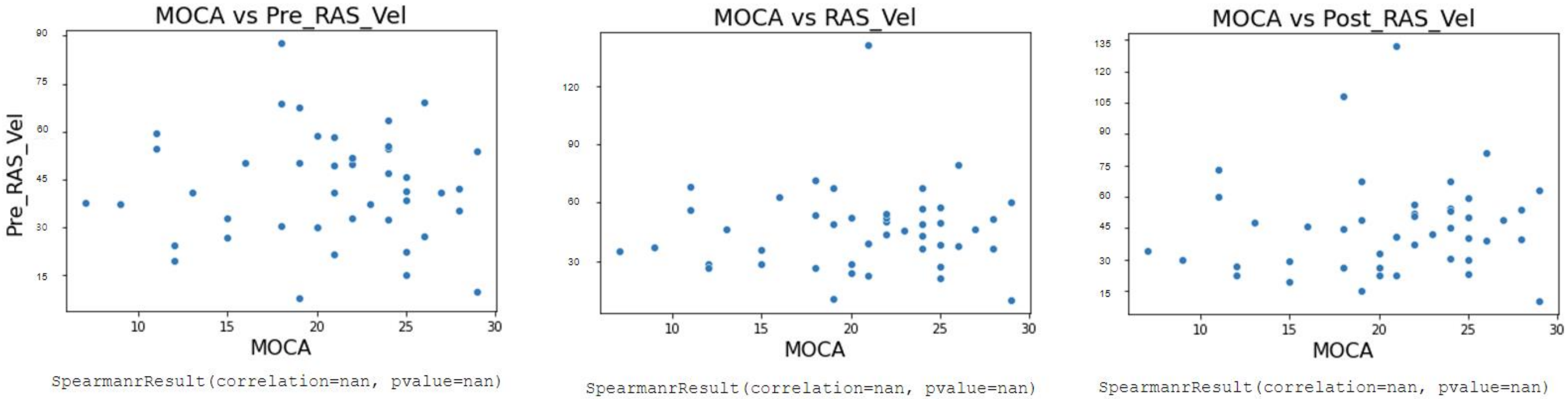

Figure 2. Associations between MoCa scores and Gait Velocity (meter/minute) in Pre, During, and Post RAS (All patients cohort, n=45)

Note: One PSP-RS participant's MoCA scores were not collected; MoCA: Montreal Cognitive Assessment; Gaitvel = gait velocity; Pre = baseline

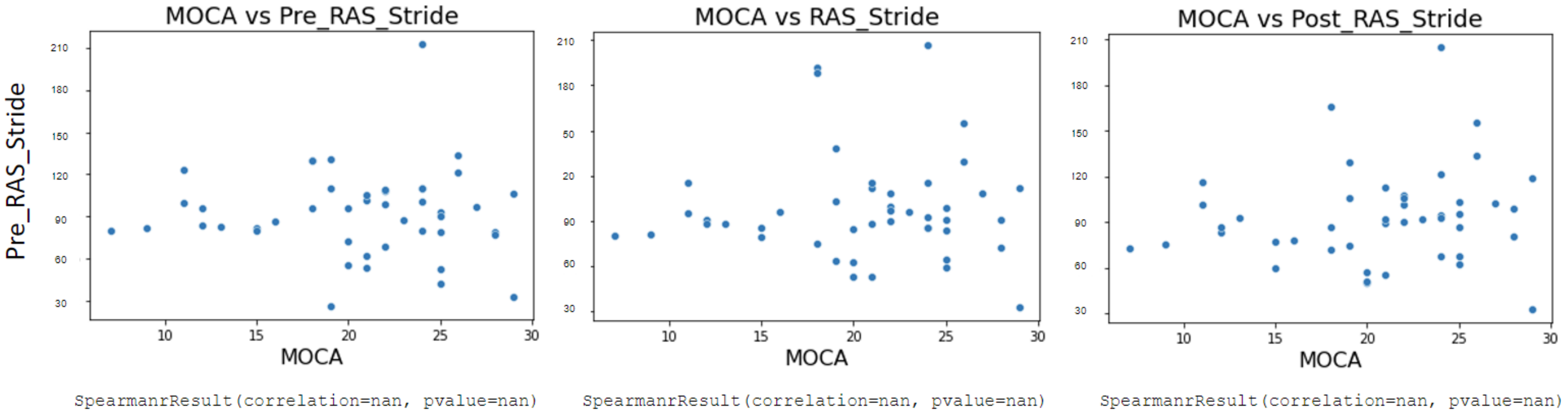

Figure 3. Associations between MoCa scores and Stride Length (cm) (All patients cohort, n=45) in Pre, During, and Post RAS

Note: One PSP-RS participant's MoCA scores were not collected; MoCA: Montreal Cognitive Assessment; Stride = Stride Length; cm = centimeters; Pre = baseline
