# Supplementary material for: Rhythmic auditory cueing in atypical parkinsonism: A pilot study
Source: Front Neurol. 2022 Oct 28;13:1018206. doi: 10.3389/fneur.2022.1018206 (PMC9650086; doi:10.3389/fneur.2022.1018206)
Supplement: Supplementary file 2 [file Table_1.DOCX]

**Table 1. Summary of Gait Parameters in All Participants and Subgroups (adjusted *p*-values)**

|  |  | Timeline | Mean (SD) | Median | SEM | Range | Comparison | *Adjusted*  *p*-value |
| --- | --- | --- | --- | --- | --- | --- | --- | --- |
| Cadence (steps/min) | All (n = 46) | Baseline | 90.40 (19.66) | 93.50 | 2.90 | 40.00-135.00 | Pre - During | .009** |
|  |  | During-RAC | 94.68 (22.23) | 96.25 | 3.28 | 33.00-130.00 | Pre - Post | .009** |
|  |  | Post-RAC | 94.73 (21.12) | 96.50 | 3.11 | 40.00-130.00 | During - Post | .960 |
|  | PSP (n = 25) | Baseline | 87.72 (20.27) | 92.00 | 4.05 | 40.00-116.00 | Pre - During | .162 |
|  |  | During-RAC | 93.98 (21.80) | 94.50 | 4.36 | 44.00-130.00 | Pre - Post | .081 |
|  |  | Post-RAC | 93.72 (19.18) | 96.00 | 3.84 | 51.00-126.00 | During - Post | .957 |
|  | PSP-RS  (n= 17) | Baseline | 83.82 (21.58) | 91.00 | 5.23 | 40.00-116.00 | Pre - During | .230 |
|  |  | During-RAC | 90.03 (22.96) | 91.00 | 5.57 | 44.00-130.00 | Pre - Post | .162 |
|  |  | Post-RAC | 89.15 (19.55) | 93.00 | 4.74 | 51.00-116.50 | During - Post | .823 |
|  | PSP-nonRS  (n = 8) | Baseline | 96.00 (15.17) | 97.25 | 5.36 | 67.00-115.00 | Pre - During | .193 |
|  |  | During-RAC | 102.38 (17.48) | 103.00 | 6.18 | 71.50-122.00 | Pre - Post | .180 |
|  |  | Post-RAC | 103.44 (15.16) | 103.50 | 5.36 | 76.00-126.00 | During - Post | .885 |
|  | CBS (n = 9) | Baseline | 94.00 (18.83) | 95.00 | 6.28 | 60.00-121.00 | Pre - During | .184 |
|  |  | During-RAC | 99.72 (22.01) | 107.00 | 3.23 | 60.00-123.00 | Pre - Post | .191 |
|  |  | Post-RAC | 101.33(21.97) | 105.00 | 7.32 | 62.00-130.00 | During - Post | .438 |
|  | MSA (n = 8) | Baseline | 89.69 (18.01) | 95.50 | 6.37 | 60.00-109.00 | Pre - During | .608 |
|  |  | During-RAC | 90.00 (28.43) | 101.25 | 10.05 | 33.00-115.00 | Pre - Post | .613 |
|  |  | Post-RAC | 90.38 (25.82) | 102.00 | 9.13 | 40.00-116.00 | During - Post | .981 |
|  | DLB (n = 4) | Baseline | 100.50 (23.90) | 93.50 | 11.95 | 80.00-135.00 | Pre - During | .865 |
|  |  | During-RAC | 97.13 (16.97) | 92.00 | 8.49 | 83.00-121.50 | Pre - Post | .280 |
|  |  | Post-RAC | 94.88 (26.71) | 92.25 | 13.35 | 65.00-130.00 | During - Post | 1 |
| Gait Velocity (m/min) | All (n = 46) | Baseline | 42.49 (17.19) | 41.45 | 2.53 | 8.08-89.00 | Pre - During | .072 |
|  |  | During-RAC | 46.05 (21.96) | 45.42 | 3.24 | 10.06-143.26 | Pre - Post | .088 |
|  |  | Post-RAC | 45.69 (23.28) | 42.37 | 3.43 | 10.36-134.11 | During - Post | .981 |
|  | PSP (n = 25) | Baseline | 43.70 (14.27) | 41.45 | 2.85 | 20.12-69.80 | Pre - During | .149 |
|  |  | During-RAC | 49.66 (24.00) | 46.33 | 4.80 | 20.12-143.26 | Pre - Post | .331 |
|  |  | Post-RAC | 47.73 (22.86) | 45.57 | 4.57 | 22.86-134.11 | During - Post | .287 |
|  | PSP-RS (n= 17) | Baseline | 39.80 (12.99) | 37.80 | 3.15 | 20.12-69.80 | Pre - During | .442 |
|  |  | During-RAC | 41.95 (12.44) | 39.93 | 3.02 | 20.12-64.01 | Pre - Post | .961 |
|  |  | Post-RAC | 39.31 (11.07) | 40.84 | 2.68 | 22.86-60.66 | During - Post | .185 |
|  | PSP-nonRS (n= 8) | Baseline | 51.97 (14.05) | 55.93 | 4.97 | 27.43-68.58 | Pre - During | .194 |
|  |  | During-RAC | 66.05 (34.22) | 61.95 | 12.10 | 28.96-143.26 | Pre - Post | .151 |
|  |  | Post-RAC | 65.65 (31.20) | 60.05 | 11.03 | 29.57-134.11 | During - Post | .988 |
|  | CBS (n = 9) | Baseline | 41.48 (19.56) | 41.45 | 6.52 | 10.06-70.41 | Pre - During | .934 |
|  |  | During-RAC | 41.36 (21.28) | 39.93 | 7.09 | 10.06-80.47 | Pre - Post | .901 |
|  |  | Post-RAC | 40.68 (22.40) | 33.53 | 7.47 | 210.36-82.30 | During - Post | .744 |
|  | MSA (n = 8) | Baseline | 34.59 (18.74) | 35.20 | 6.63 | 8.08-55.63 | Pre - During | .175 |
|  |  | During-RAC | 38.79 (18.43) | 44.20 | 5.52 | 10.67-61.26 | Pre - Post | .162 |
|  |  | Post-RAC | 41.05 (18.57) | 46.56 | 6.57 | 15.09-64.01 | During - Post | .138 |
|  | DLB (n = 4) | Baseline | 53.00 (25.15) | 44.81 | 12.57 | 33.38-89.00 | Pre - During | .844 |
|  |  | During-RAC | 48.54 (17.37) | 42.90 | 8.69 | 35.66-72.69 | Pre - Post | .984 |
|  |  | Post-RAC | 53.42 (39.47) | 42.06 | 19.74 | 19.81-109.73 | During - Post | 1 |
| Stride Length (cm) | All (n = 46) | Baseline | 93.96 (31.22) | 96.01 | 4.60 | 26.82-215.80 | Pre - During | .418 |
|  |  | During-RAC | 99.27 (34.84) | 92.05 | 5.14 | 33.53-209.70 | Pre - Post | 1 |
|  |  | Post-RAC | 94.73 (31.64) | 92.23 | 4.67 | 33.41-207.87 | During - Post | .497 |
|  | PSP (n = 25) | Baseline | 100.49 (30.00) | 94.49 | 6.00 | 62.79-215.80 | Pre - During | .403 |
|  |  | During-RAC | 106.19 (32.34) | 97.54 | 6.47 | 73.76-209.70 | Pre - Post | .844 |
|  |  | Post-RAC | 98.97 (27.64) | 93.27 | 5.53 | 27.64-207.87 | During - Post | .176 |
|  | PSP-RS (n= 17) | Baseline | 97.57 (34.33) | 89.00 | 8.33 | 62.79-215.80 | Pre - During | .620 |
|  |  | During-RAC | 104.28 (37.56) | 92.05 | 9.11 | 73.76-209.70 | Pre - Post | .596 |
|  |  | Post-RAC | 94.12 (30.70) | 88.39 | 7.45 | 68.28-207.87 | During - Post | .171 |
|  | PSP-nonRS (n = 8) | Baseline | 106.68 (18.08) | 109.12 | 6.39 | 81.69-132.89 | Pre - During | .482 |
|  |  | During-RAC | 110.26 (18.21) | 113.39 | 6.44 | 81.08-140.21 | Pre - Post | .711 |
|  |  | Post-RAC | 108.97 (17.11) | 111.25 | 6.05 | 78.03-131.67 | During - Post | .798 |
|  | CBS (n = 9) | Baseline | 84.87 (32.63) | 97.54 | 10.88 | 33.53-135.33 | Pre - During | .169 |
|  |  | During-RAC | 78.57 (28.81) | 85.95 | 9.60 | 33.53-131.67 | Pre - Post | .466 |
|  |  | Post-RAC | 77.37 (33.80) | 63.40 | 11.27 | 33.41-135.94 | During - Post | .963 |
|  | MSA (n = 8) | Baseline | 79.78 (34.48) | 90.22 | 12.19 | 26.82-123.14 | Pre - During | .434 |
|  |  | During-RAC | 91.97 (33.28) | 89.61 | 11.76 | 54.25-157.28 | Pre - Post | .162 |
|  |  | Post-RAC | 97.27 (32.19) | 98.15 | 11.40 | 56.39-157.89 | During - Post | .144 |
|  | DLB (n = 4) | Baseline | 101.96 (24.16) | 97.54 | 12.08 | 81.08-131.67 | Pre - During | .823 |
|  |  | During-RAC | 117.20 (52.85) | 96.01 | 26.43 | 81.69-195.07 | Pre - Post | .964 |
|  |  | Post-RAC | 102.87 (47.85) | 91.14 | 23.92 | 60.96-168.25 | During - Post | .450 |

Note: **p* < .05, ***p* < .01, ****p* < .001; SD *=* standard deviation; SEM: standard error of the mean

**Table 2. Gait Parameters for TAU and SYN**

|  |  | Timeline | Mean (*SD*) | Median | SEM | Range | Comparison | *p*-value |
| --- | --- | --- | --- | --- | --- | --- | --- | --- |
| Cadence (steps/min) | TAU (n = 34) | Baseline | 89.38 (19.82) | 93.50 | 3.40 | 40.00-121.00 | Pre - During | **.00039***** |
|  |  | During-RAS | 95.50 (21.67) | 96.25 | 3.72 | 44.00-130.00 | Pre - Post | .**00024***** |
|  |  | Post-RAS | 95.74 (19.91) | 96.50 | 3.41 | 51.00-130.00 | During - Post | .771 |
|  | SYN (n = 12) | Baseline | 93.29 (19.76) | 93.50 | 5.70 | 60.00-135.00 | Pre - During | .791 |
|  |  | During-RAS | 92.38 (24.60) | 96.25 | 7.10 | 33.00-121.50 | Pre - Post | .838 |
|  |  | Post-RAS | 91.88 (24.97) | 96.75 | 7.21 | 40.00-130.00 | During - Post | .970 |
| Gait Velocity (m/min) | TAU (n = 34) | Baseline | 43.11 (15.55) | 41.45 | 2.67 | 10.06-70.41 | Pre - During | .038* |
|  |  | During-RAS | 47.46 (23.29) | 45.42 | 4.00 | 10.06-143.26 | Pre - Post | .161 |
|  |  | Post-RAS | 45.87 (22.62) | 42.37 | 3.88 | 10.36-134.11 | During - Post | .326 |
|  | SYN (n = 12) | Baseline | 40.73 (21.87) | 40.54 | 6.31 | 8.08- 89.00 | Pre - During | .339 |
|  |  | During-RAS | 42.04 (26.11) | 44.04 | 5.18 | 10.67-72.69 | Pre - Post | .130 |
|  |  | Post-RAS | 45.17 (26.11) | 44.81 | 7.54 | 15.09-109.73 | During - Post | .110 |
| Stride Length (cm) | TAU (n = 34) | Baseline | 96.35 (31.01) | 96.01 | 5.32 | 33.53-215.80 | Pre - During | .822 |
|  |  | During-RAS | 98.88 (33.39) | 92.05 | 5.72 | 33.53-209.70 | Pre - Post | .301 |
|  |  | Post-RAS | 93.18 (30.42) | 91.01 | 5.22 | 33.41-207.87 | During - Post | .104 |
|  | SYN (n = 12) | Baseline | 87.17 (32.17) | 91.74 | 9.29 | 26.82-131.67 | Pre - During | .064 |
|  |  | During-RAS | 100.38 (40.26) | 89.91 | 11.62 | 54.25-195.07 | Pre - Post | .151 |
|  |  | Post-RAS | 99.13 (35.93) | 98.15 | 10.37 | 56.39-168.25 | During - Post | .519 |

**Table 3. Delta in Gait Parameters for TAU vs SYN**

|  |  | TAU | | | | SYN | | | |  |
| --- | --- | --- | --- | --- | --- | --- | --- | --- | --- | --- |
|  |  | Mean (*SD*) | Median | *SEM* | Range | Mean (*SD*) | Median | *SEM* | Range | *p*-value |
| Cadence (steps/min) | $\Delta$ *pre-during* | 6.11 (7.92) | 6.00 | 1.36 | -10.00-22.00 | -0.92 (10.41) | 3.50 | 3.00 | -27.00-7.50 | .023* |
|  | $\Delta$ *during-post* | 0.24 (6.89) | 1.75 | 1.18 | -13.50-20.00 | -0.50 (6.92) | 0.00 | 2.00 | -18.00-8.50 | .495 |
|  | $\Delta$ *pre-post* | 6.35 (8.17) | 8.00 | 1.40 | -13.00-30.00 | -1.42 (8.74) | 0.00 | 2.52 | -20.00-11.00 | .007** |
| Gait Velocity (m/min) | $\Delta$ *pre-during* | 4.35 (15.42) | 1.52 | 2.64 | -15.16-84.12 | 1.31 (7.39) | 2.51 | 2.13 | -16.31-10.67 | .435 |
|  | $\Delta$ *during-post* | -1.60 (5.79) | 0.15 | 0.99 | -19.51-6.32 | 3.14 (12.02) | 1.68 | 3.47 | -16.30-37.03 | .073 |
|  | $\Delta$ *pre-post* | 2.76 (15.43) | 2.13 | 2.65 | -26.21-74.98 | 4.45 (9.01) | 4.80 | 2.60 | -13.56-20.73 | .155 |
| Stride Length (cm) | $\Delta$ *pre-during* | 2.53 (16.81) | 0.00 | 2.88 | -21.34-59.74 | 13.21 (22.32) | 4.88 | 6.44 | -15.24-63.40 | .056 |
|  | $\Delta$ *during-post* | -5.71 (19.62) | -1.83 | 3.36 | -103.02-17.68 | -1.24 (12.72) | 2.59 | 3.67 | -26.82-10.97 | .079 |
|  | $\Delta$ *pre-post* | -3.18 (15.16) | -0.91 | 2.60 | -45.72-30.48 | 11.96 (21.35) | 7.92 | 6.16 | -22.56-48.77 | .023* |

|  |  |  | Mean (*SD*) | Median | *SEM* | Range | Comparison | *p*-value |
| --- | --- | --- | --- | --- | --- | --- | --- | --- |
| All Patients (n = 46) | Cadence (steps/min) | $\Delta$ *pre-during* | 4.28 (9.07) | 5.00 | 1.34 | -27.00-22.00 | $\Delta$ *pre-during vs* $\Delta$ *during-post* | .016* |
|  |  | $\Delta$ *during-post* | 0.04 (6.83) | 5.00 | 1.01 | -20.00-30.00 | $\Delta$ *pre-during vs* $\Delta$ *pre-post* | .747 |
|  |  | $\Delta$ *pre-post* | 4.33 (8.91) | 1.25 | 1.31 | -18.00-20.00 | $\Delta$ *post-during vs* $\Delta$ *pre-post* | .001** |
|  | Gait Velocity (m/min) | $\Delta$ *pre-during* | 3.56 (13.77) | 1.98 | 6.66 | -16.31-84.12 | $\Delta$ *pre-during vs* $\Delta$ *during-post* | .027* |
|  |  | $\Delta$ *during-post* | -0.36 (8.02) | 0.46 | 3.88 | -19.51-37.03 | $\Delta$ *pre-during vs* $\Delta$ *pre-post* | .981 |
|  |  | $\Delta$ *pre-post* | 3.20 (13.96) | 2.36 | 6.75 | -26.21-74.98 | $\Delta$ *post-during vs* $\Delta$ *pre-post* | .024* |
|  | Stride Length (cm) | $\Delta$ *pre-during* | 5.31 (18.75) | 0.91 | 0.05 | -21.34-63.40 | $\Delta$ *pre-during vs* $\Delta$ *during-post* | .106 |
|  |  | $\Delta$ *during-post* | -4.54 (18.05) | -0.37 | 0.04 | -103.02-17.69 | $\Delta$ *pre-during vs* $\Delta$ *pre-post* | .331 |
|  |  | $\Delta$ *pre-post* | 0.77 (18.03) | -0.37 | 0.04 | -45.72-48.77 | $\Delta$ *post-during vs* $\Delta$ *pre-post* | .232 |
| PSP (n = 25) | Cadence (steps/min) | $\Delta$ *pre-during* | 6.26 (8.09) | 6.00 | 1.62 | -10.00-22.00 | $\Delta$ *pre-during vs* $\Delta$ *during-post* | .003** |
|  |  | $\Delta$ *during-post* | -0.26 (7.75) | -2.00 | 1.55 | -13.50-20.00 | $\Delta$ *pre-during vs* $\Delta$ *pre-post* | .833 |
|  |  | $\Delta$ *pre-post* | 6.00 (8.60) | 7.00 | 1.72 | -13.00-30.00 | $\Delta$ *post-during vs* $\Delta$ *pre-post* | .012* |
|  | Gait Velocity (m/min) | $\Delta$ *pre-during* | 5.96 (17.54) | 3.96 | 11.51 | -15.16-84.12 | $\Delta$ *pre-during vs* $\Delta$ *during-post* | .013* |
|  |  | $\Delta$ *during-post* | -1.93 (5.17) | 0.00 | 3.39 | -17.37-5.18 | $\Delta$ *pre-during vs* $\Delta$ *pre-post* | .117 |
|  |  | $\Delta$ *pre-post* | 4.04 (16.70) | 2.13 | 10.96 | -24.23-74.98 | $\Delta$ *post-during vs* $\Delta$ *pre-post* | .022* |
|  | Stride Length (cm) | $\Delta$ *pre-during* | 5.71 (18.14) | 4.88 | .06 | -21.34-59.74 | $\Delta$ *pre-during vs* $\Delta$ *during-post* | .179 |
|  |  | $\Delta$ *during-post* | -7.32 (21.28) | -3.05 | .07 | -103.02-7.92 | $\Delta$ *pre-during vs* $\Delta$ *pre-post* | .039* |
|  |  | $\Delta$ *pre-post* | -1.62 (14.79) | -1.22 | .05 | -43.28-30.48 | $\Delta$ *post-during vs* $\Delta$ *pre-post* | .120 |
| PSP_RS (n=17) | Cadence (steps/min) | $\Delta$ *pre-during* | 6.21 (8.41) | 7.50 | 2.04 | -10.00-19.50 | $\Delta$ *pre-during vs* $\Delta$ *during-post* | .051 |
|  |  | $\Delta$ *during-post* | -0.88 (8.26) | -2.00 | 2.00 | -13.50-20.00 | $\Delta$ *pre-during vs* $\Delta$ *pre-post* | 0.678 |
|  |  | $\Delta$ *pre-post* | 5.32 (7.80) | 7.00 | 1.89 | -13.00-17.50 | $\Delta$ *post-during vs* $\Delta$ *pre-post* | .017** |
|  | Gait Velocity (m/min) | $\Delta$ *pre-during* | 2.15 (7.71) | 1.52 | 6.13 | -15.16-12.80 | $\Delta$ *pre-during vs* $\Delta$ *during-post* | .080 |
|  |  | $\Delta$ *during-post* | -2.64 (5.48) | -1.83 | 4.36 | -17.37-3.35 | $\Delta$ *pre-during vs* $\Delta$ *pre-post* | .065 |
|  |  | $\Delta$ *pre-post* | -.50 (8.44) | 1.83 | 6.71 | -24.23-14.33 | $\Delta$ *post-during vs* $\Delta$ *pre-post* | .221 |
|  | Stride Length (cm) | $\Delta$ *pre-during* | 6.71 (21.70) | 4.88 | 0.09 | -21.34-59.74 | $\Delta$ *pre-during vs* $\Delta$ *during-post* | .243 |
|  |  | $\Delta$ *during-post* | -10.16 (25.33) | -3.05 | 0.10 | -103.02-7.92 | $\Delta$ *pre-during vs* $\Delta$ *pre-post* | .057 |
|  |  | $\Delta$ *pre-post* | -3.46 (17.16) | -6.10 | 0.07 | -43.28-30.48 | $\Delta$ *post-during vs* $\Delta$ *pre-post* | .379 |
| PSP_nonRS | Cadence (steps/min) | $\Delta$ *pre-during* | 6.38 (7.92) | 5.25 | 2.80 | -5.00-22.00 | $\Delta$ *pre-during vs* $\Delta$ *during-post* | .125 |
|  |  | $\Delta$ *during-post* | 1.06 (6.86) | 1.25 | 2.43 | -8.00-10.00 | $\Delta$ *pre-during vs* $\Delta$ *pre-post* | .641 |
|  |  | $\Delta$ *pre-post* | 7.44 (10.55) | 5.75 | 3.73 | -5.00-30.00 | $\Delta$ *post-during vs* $\Delta$ *pre-post* | .055 |
|  | Gait Velocity (m/min) | $\Delta$ *pre-during* | 14.08 (28.44) | 4.88 | 32.99 | 0.00-84.12 | $\Delta$ *pre-during vs* $\Delta$ *during-post* | .018 |
|  |  | $\Delta$ *during-post* | -0.40 (4.36) | 0.30 | 5.06 | -9.14-5.18 | $\Delta$ *pre-during vs* $\Delta$ *pre-post* | .933 |
|  |  | $\Delta$ *pre-post* | 13.68 (25.25) | 5.33 | 29.29 | -0.91-74.98 | $\Delta$ *post-during vs* $\Delta$ *pre-post* | .018 |
|  | Stride Length (cm) | $\Delta$ *pre-during* | 3.58 (6.63) | 5.18 | 0.04 | -8.53-10.97 | $\Delta$ *pre-during vs* $\Delta$ *during-post* | .195 |
|  |  | $\Delta$ *during-post* | -1.30 (5.00) | -2.44 | 0.03 | -8.53-6.71 | $\Delta$ *pre-during vs* $\Delta$ *pre-post* | .547 |
|  |  | $\Delta$ *pre-post* | 2.29 (7.15) | 2.13 | 0.04 | -7.92-11.59 | $\Delta$ *post-during vs* $\Delta$ *pre-post* | .250 |
| CBS (n = 9) | Cadence (steps/min) | $\Delta$ *pre-during* | 5.72 (7.90) | 6.00 | 2.63 | -6.50-7.00 | $\Delta$ *pre-during vs* $\Delta$ *during-post* | .359 |
|  |  | $\Delta$ *during-post* | 1.61 (3.56) | 2.50 | 1.19 | -5.00-7.00 | $\Delta$ *pre-during vs* $\Delta$ *pre-post* | .203 |
|  |  | $\Delta$ *pre-post* | 7.33 (7.18) | 10.00 | 2.39 | -4.00-17.00 | $\Delta$ *post-during vs* $\Delta$ *pre-post* | .068 |
|  | Gait Velocity (m/min) | $\Delta$ *pre-during* | -0.12 (5.16) | -1.37 | 5.64 | -6.71-10.06 | $\Delta$ *pre-during vs* $\Delta$ *during-post* | 1 |
|  |  | $\Delta$ *during-post* | -0.68 (7.53) | 1.83 | 8.24 | -19.51-6.32 | $\Delta$ *pre-during vs* $\Delta$ *pre-post* | .496 |
|  |  | $\Delta$ *pre-post* | -0.80 (11.20) | 0.30 | 12.25 | -26.21-11.89 | $\Delta$ *post-during vs* $\Delta$ *pre-post* | .778 |
|  | Stride Length (cm) | $\Delta$ *pre-during* | -6.30 (7.72) | -4.88 | 0.04 | -18.29-6.71 | $\Delta$ *pre-during vs* $\Delta$ *during-post* | .426 |
|  |  | $\Delta$ *during-post* | -1.21 (14.09) | 1.22 | 0.08 | -34.14-17.68 | $\Delta$ *pre-during vs* $\Delta$ *pre-post* | .820 |
|  |  | $\Delta$ *pre-post* | -7.50 (16.23) | -0.61 | 0.09 | -45.72-9.75 | $\Delta$ *post-during vs* $\Delta$ *pre-post* | .050 |
| MSA (n = 8) | Cadence (steps/min) | $\Delta$ *pre-during* | 0.31 (11.73) | 4.75 | 4.15 | -27.00-7.50 | $\Delta$ *pre-during vs* $\Delta$ *during-post* | .641 |
|  |  | $\Delta$ *during-post* | 0.38 (3.99) | 0.00 | 1.41 | -4.50-7.00 | $\Delta$ *pre-during vs* $\Delta$ *pre-post* | .945 |
|  |  | $\Delta$ *pre-post* | 0.69 (9.28) | 2.50 | 3.28 | -20.00-11.00 | $\Delta$ *post-during vs* $\Delta$ *pre-post* | .383 |
|  | Gait Velocity (m/min) | $\Delta$ *pre-during* | 4.19 (5.32) | 4.34 | 6.17 | -5.64-10.67 | $\Delta$ *pre-during vs* $\Delta$ *during-post* | .312 |
|  |  | $\Delta$ *during-post* | 2.27 (2.00) | 1.98 | 2.32 | -0.30-5.64 | $\Delta$ *pre-during vs* $\Delta$ *pre-post* | .023* |
|  |  | $\Delta$ *pre-post* | 6.46 (4.87) | 7.47 | 5.65 | 0.00-12.19 | $\Delta$ *post-during vs* $\Delta$ *pre-post* | .055 |
|  | Stride Length (cm) | $\Delta$ *pre-during* | 12.19 (18.15) | 9.14 | 0.11 | -15.24-37.80 | $\Delta$ *pre-during vs* $\Delta$ *during-post* | .461 |
|  |  | $\Delta$ *during-post* | 5.30 (3.87) | 4.88 | 0.02 | 0.61-10.97 | $\Delta$ *pre-during vs* $\Delta$ *pre-post* | .008** |
|  |  | $\Delta$ *pre-post* | 17.49 (18.44) | 16.46 | 0.11 | -6.10-48.77 | $\Delta$ *post-during vs* $\Delta$ *pre-post* | .109 |
| DLB (n=4) | Cadence (steps/min) | $\Delta$ *pre-during* | -3.38 (7.97) | -1.50 | 3.99 | -13.50-3.00 | $\Delta$ *pre-during vs* $\Delta$ *during-post* | .625 |
|  |  | $\Delta$ *during-post* | -2.25 (11.51) | 0.25 | 5.75 | -18.00-8.50 | $\Delta$ *pre-during vs* $\Delta$ *pre-post* | 1.00 |
|  |  | $\Delta$ *pre-post* | -5.63 (6.57) | -3.75 | 3.29 | -15.00-0.00 | $\Delta$ *post-during vs* $\Delta$ *pre-post* | .875 |
|  | Gait Velocity (m/min) | $\Delta$ *pre-during* | -4.46 (8.24) | -2.13 | 13.52 | -16.31-2.74 | $\Delta$ *pre-during vs* $\Delta$ *during-post* | .625 |
|  |  | $\Delta$ *during-post* | 4.88 (22.68) | -0.61 | 37.21 | -16.31-37.03 | $\Delta$ *pre-during vs* $\Delta$ *pre-post* | 1.00 |
|  |  | $\Delta$ *pre-post* | 0.42 (14.50) | -2.74 | 23.79 | -13.56-20.73 | $\Delta$ *post-during vs* $\Delta$ *pre-post* | .375 |
|  | Stride Length (cm) | $\Delta$ *pre-during* | 15.25 (32.40) | 2.13 | 0.27 | -6.71-63.40 | $\Delta$ *pre-during vs* $\Delta$ *during-post* | .375 |
|  |  | $\Delta$ *during-post* | -14.33 (14.72) | -16.76 | 0.12 | -26.82-3.05 | $\Delta$ *pre-during vs* $\Delta$ *pre-post* | .250 |
|  |  | $\Delta$ *pre-post* | 0.91 (25.17) | -5.18 | 0.21 | -22.56-36.58 | $\Delta$ *post-during vs* $\Delta$ *pre-post* | .625 |

**Table 4. Effect sizes and sample size calculations**

- Two-tailed Wilcoxon Signed-rank test (matched pairs)
- Types of power analysis: A priori: Compute required sample size – given alpha power, and effect size
- α error probability= 0.05
- Power (1- **β** error probability) = 0.80

| **Gait Parameters** | **Comparison** | **P-value** | **Effect size (d)** | **Estimated total sample size** |
| --- | --- | --- | --- | --- |
| Cadence | Pre vs Post | 0.002 | 0.212359 | 185 |
|  | Pre vs During | 0.001 | 0.204345 | 199 |
|  | During vs Post | 0.747 | 0.002307 | 1,544,332 |
| Velocity | Pre vs Post | 0.038 | 0.158142 | 331 |
|  | Pre vs During | 0.024 | 0.181865 | 251 |
|  | During vs Post | 0.981 | -0.01592 | 32,433 |
| Stride length | Pre vs Post | 0.926 | 0.024499 | 13,697 |
|  | Pre vs During | 0.232 | 0.160763 | 321 |
|  | During vs Post | 0.331 | -0.13658 | 443 |
